# Pan-cerebral sodium elevations in vascular dementia: Evidence for disturbed brain-sodium homeostasis

**DOI:** 10.3389/fnagi.2022.926463

**Published:** 2022-07-18

**Authors:** Sasha A. Philbert, Jingshu Xu, Stephanie J. Church, Richard D. Unwin, Federico Roncaroli, Garth J. S. Cooper

**Affiliations:** ^1^Division of Cardiovascular Sciences, Centre for Advanced Discovery and Experimental Therapeutics, Faculty of Biology, Medicine and Health, School of Medical Sciences, The University of Manchester, Manchester Academic Health Science Centre, Manchester, United Kingdom; ^2^Division of Cancer Sciences, Stoller Biomarker Discovery Centre, Faculty of Biology, Medicine and Health, School of Medical Sciences, The University of Manchester, Manchester, United Kingdom; ^3^Division of Neuroscience and Experimental Psychology, Geoffrey Jefferson Brain Research Centre, Faculty of Biology, Medicine and Health, School of Biological Sciences, The University of Manchester, Manchester, United Kingdom; ^4^Faculty of Science, School of Biological Sciences, The University of Auckland, Auckland, New Zealand

**Keywords:** vascular dementia, metal dyshomeostasis, brain-copper levels, Na/K ratio, brain-sodium levels, mass spectrometry, brain-potassium levels, Na^+^/K^+^-exchanging ATPase

## Abstract

Vascular dementia (VaD) is the second most common cause of cognitive impairment amongst the elderly. However, there are no known disease-modifying therapies for VaD, probably due to incomplete understanding of the molecular basis of the disease. Despite the complex etiology of neurodegenerative conditions, a growing body of research now suggests the potential involvement of metal dyshomeostasis in the pathogenesis of several of the age-related dementias. However, by comparison, there remains little research investigating brain metal levels in VaD. In order to shed light on the possible involvement of metal dyshomeostasis in VaD, we employed inductively coupled plasma-mass spectrometry to quantify the levels of essential metals in *post-mortem* VaD brain tissue (*n* = 10) and age-/sex-matched controls (*n* = 10) from seven brain regions. We found novel evidence for elevated wet-weight cerebral sodium levels in VaD brain tissue in six out of the seven regions analyzed. Decreased cerebral-potassium levels as well as increased Na/K ratios (consistent with high tissue sodium and low potassium levels) were also observed in several brain regions. These data suggest that reduced Na^+^/K^+^-exchanging ATPase (EC 7.2.2.13) activity could contribute to the contrasting changes in sodium and potassium measured here.

## Introduction

Vascular dementia (VaD) comprises a spectrum of conditions characterized by cerebrovascular disease that leads to or causes cognitive impairment, behavioral abnormalities, and motor dysfunction (Looi and Sachdev, 1999; Bazner et al., 2000; Sachdev et al., 2004). So defined, VaD accounts for about 20% of all cases of age-related dementia (Rizzi et al., 2014). The clinical diagnosis of VaD can be challenging, because signs and symptoms can overlap with other neurodegenerative conditions such as Alzheimer’s disease (AD; O’Brien and Thomas, 2015). Diabetes, hypertension, obesity, and stroke are also predisposing factors. The pathological changes that lead to VaD include small vessel disease secondary to arteriosclerosis, and amyloid angiopathy (Deramecourt et al., 2012). Small vessel disease often co-exists with AD pathology, highlighting the similarity between the two conditions (Jellinger and Attems, 2005; Deramecourt et al., 2012). Despite considerable advances in the understanding of the pathophysiology of VaD, the key molecular mechanisms underlying the disease remain to be identified. Therapeutic options are largely restricted to repurposing medications used in AD, but this strategy offers limited clinical benefit (Sun, 2018).

Essential metals play key roles in human physiology, often due to their functions as necessary cofactors in key enzymatic reactions (Lothian et al., 2013). However, they can also cause cellular dysfunction if concentrations are maintained outside their normal physiological limits (Fraga, 2005). A growing body of evidence implies that significantly perturbed levels of several essential metals occur in neurodegenerative diseases such as AD (Xu et al., 2017), Parkinson’s disease dementia (PDD; Scholefield et al., 2021), and amyotrophic lateral sclerosis (Violi et al., 2020). By contrast, relatively little is known about the metallome in VaD. Some studies have reported increased brain-iron deposition in VaD patients using clinical imaging techniques (Liem et al., 2012; De Reuck et al., 2014; Liu et al., 2015; Moon et al., 2016; Sun et al., 2017). Due to the nature of these methods, not all metals can be analyzed *in vivo*, which limits the scope of such investigations. There is evidence that impaired homeostatic regulation of zinc and copper may play substantive roles in the pathogenesis of VaD (Kawahara et al., 2014; Tanaka and Kawahara, 2017). However, the evidence in these reports was based on neuronal models, which may not accurately reflect the pattern of metal perturbation in human VaD.

To investigate potential metal dyshomeostasis in human VaD, our group initially measured metal concentrations in *post-mortem* hippocampal tissue from patients and matched controls. This preliminary study found strong evidence for elevated sodium and lowered potassium levels, which alterations were postulated to be due to dysfunction of the Na^+^/K^+^-exchanging ATPase secondary to underlying impairment of metabolic energy (ATP) production (Philbert et al., 2022). A comprehensive multiregional assessment of metal concentrations in VaD *post-mortem* brains was therefore required to determine the extent and severity of these defects throughout the human brain.

In the present study, we used inductively coupled plasma-mass spectrometry (ICP-MS) to measure the concentrations of eight essential metals (Na, Mg, K, Ca, Mn, Fe, Cu, and Zn) and the metalloid, Se, in human *post-mortem* tissue from six brain regions from cases with small vessel disease causing VaD and non-demented age- and sex-matched controls. Given the findings from our preliminary study using hippocampal tissue (Philbert et al., 2022), our research hypothesis was that increased Na and decreased K would be evident in severely affected regions in VaD to levels comparable to that of the hippocampus. Here, we found wet- and dry-weight Na/K ratios to be significantly increased in several regions as well as widespread cerebral-sodium elevation in VaD cases compared with controls. These data imply probable decreases in the activity of the mitochondrial sodium-potassium pump (Na^+^/K^+^-ATPase) probably in response to cerebral energy deficiency, which could exacerbate the neuropathology of VaD.

## Materials and methods

### Ethics approval

All experiments were performed in accordance with the relevant United Kingdom and international guidelines and regulations as stated below. The study of *post-mortem* VaD/control tissue received Local Research Ethics Committee approval supplied by the Southwest Dementia Brain Bank (SWDBB). Informed consent for the collection of tissue was obtained by the SWDBB. Consent for collection of AD cases was as previously stated (Xu et al., 2017).

### Case selection

Brain tissues from the 10 VaD cases, all of whom had a confirmed neuropathological diagnosis of small vessel disease at *post-mortem* examination, and 10 age-/sex-matched controls were obtained from the SWDBB. Sets of tissue from each of the thalamus (TH), basal ganglia (BG), cingulate gyrus (CG), frontal gyrus (FG), middle temporal gyrus (MTG), occipital cortex (OC), and hippocampus (HP) were acquired. These regions were selected because they are commonly affected in VaD; additionally, they allow for direct comparisons with our previous metallomic studies in AD (Xu et al., 2017). Although cerebrovascular damage is known to be evident in the hindbrain of VaD patients, analysis of the hindbrain region could not be performed because of insufficient tissue availability. Mean *post-mortem* delay was <35 h for both VaD case and control groups; importantly, extended *post-mortem* delay of up to 72 h has been not to interfere with brain-metal levels (Scholefield et al., 2020). The sampling of *post-mortem* VaD tissue from multiple regions was also postulated to allow both the identification of inter-regional differences in VaD, and to enable informative comparisons with metallomic data from our prior age-related datasets including AD, Huntington’s disease (HD), and Parkinson’s disease dementia (PDD).

The inclusion criteria for VaD cases included: confirmed neuropathological diagnosis of VaD at *post-mortem* examination; AD Braak stage of 3 or less (although only case 1105 had a Braak stage of 3); non-amyloid small vessel disease; mild to moderate cerebral amyloid angiopathy (CAA); histopathological evidence of microinfarction; and no histopathological evidence of other neurological diseases likely to cause dementia. However, due to the limited sample metadata, complete fulfillment of these criteria could not be confirmed for all cases (e.g., cause of death and Braak stage were not specified for some cases). Despite this, all cases manifested evidence of ischemic cerebral damage, a confirmed diagnosis of VaD at *post-mortem* examination (as specified by the SWDBB), and no histopathological evidence of other neurological diseases likely to cause dementia. Although mild to moderate CAA was requested, case 1105 was found to have severe CAA potentially consistent with mixed dementia. However, this case did not present any outlying metal values and received a clinical and *post-mortem* diagnosis of VaD which provided adequate justification for its inclusion in this study. Controls had no clinical history of dementia or other brain disease, and no other neurological abnormalities.

The Montreal Cognitive Assessment (MoCA), which is considered to be the preferred neuropsychometric test for the assessment of VaD (Freitas et al., 2012), was recorded in eight cases. It should also be noted that the cause of death for patient 1008 was listed as VaD and type 2 diabetes (T2D; Supplementary Table 1). Although T2D is a prominent risk factor for VaD, it can also itself lead directly to dementia and cognitive impairment.

Metal values from the HP in the present study were taken from our group’s previous preliminary investigation of VaD *post-mortem* tissue (Philbert et al., 2022). However, due to tissue limitations, the same VaD cohort that was used for our group’s previous investigation of VaD HP tissue (Supplementary Table 1; Philbert et al., 2022) could not be obtained for the remaining regions that were analyzed in this study. Group characteristics for VaD cases and controls are as shown in Table 1 (excluding the hippocampal cohort from the previous study) and individual patient characteristics, including age and *post-mortem* delay are documented in Supplementary Tables 1, 2. Group characteristics for the hippocampal cohort are shown in Supplementary Table 3.

**TABLE 1:** Group characteristics excluding the hippocampal cohort.

Variable	Control	VaD
Number	10	10
Age	82 (9)	84 (8)
Male sex, *n* (%)	4 (40)	4 (40)
*Post-mortem* delay (h)	34.65 (6.27)	34.08 (12.44)
Brain wt (g)	1214 (114)	1221 (120)
Water content (%)	81.3 (1.46)	81.4 (2.39)
Wet-wt/dry-wt	5.56 (0.39)	5.67 (0.72)

Values are: age, post-mortem delay, brain wt and water content, mean (SD); wet-wt/dry-wt ratio, mean (SD) averaged across all samples. All differences were non-significant.

### Tissue dissection

All samples were transported from the SWDBB to our laboratory on dry ice and then stored at −80°C. Prior to analysis, two sets of aliquots were prepared for dry- and separate wet-weight analysis; both analyses were performed to enable comparisons with other literature reports. Samples were thawed briefly on ice before dissection. Wet-weight aliquots of 50 ± 5 mg were dissected using a ceramic scalpel to avoid metal contamination and placed into 2-mL micro centrifuge tubes. For dry-weight analysis, 50 ± 5 mg of the same sample was dissected as previously described and dried to constant weight in a centrifugal concentrator (Savant SpeedVac; Thermo Fisher Scientific, Waltham, MA, United States) as further described below.

### Digestion

Before digestion, all samples were briefly centrifuged at 2400 × *g* (Heraeus Pico 17 Centrifuge; Thermo Fisher Scientific, Waltham, MA, United States) to ensure that the tissue sat at the bottom of the tubes. Concentrated nitric acid (A509 Trace Metal Grade; Fisher, Loughborough, United Kingdom) and 5% Agilent Internal Standard mixture (5183-4681; Agilent Technologies, Cheadle, United Kingdom) were used to make the tissue digestion mixture. Calibration standards were prepared to the appropriate dilutions (Supplementary Table 4) using an Environmental Calibration Standard Mixture (Agilent 5189-4688) and 2% nitric acid digestion mix. For both wet- and dry-weight analysis, 200 mL of digestion mix was added to each sample including two empty 2-mL microcentrifuge tubes for use as digestion blanks. All microcentrifuge tube lids were punctured using a septum remover to prevent pressure build up before being transferred into a Dri-Block DB3 heater (Techne, Staffordshire, United Kingdom) at room temperature. The temperature was set to 60°C for 30 min and then increased to 100°C for a further 3.5 h. After the digestion procedure, 100 μL of each sample or digestion blank was added to 5 mL of LC/MS grade water in 15-mL centrifuge tubes (Greiner). Samples were retained at room temperature pending ICP-MS analysis.

### Inductively coupled plasma-mass spectrometry

Due to differences in the reporting of either wet- or dry-weight ICP-MS analysis in the literature, we have employed both methods to ensure the consistency of data for between-study comparisons. However, as the coefficient of variation (CV) values for wet-weight ICP-MS analysis were moderately lower than corresponding dry-weight values (Supplementary Tables 5–7), we have initially reported wet-weight elemental concentrations, which reflect the actual physiological metal levels in the tissues, followed by the dry-weight concentrations in the Supplementary Tables 8–14. In the present study, we used the same protocol for ICP-MS measurement as in our previous case-control investigations of *post-mortem* brain tissue in AD (Xu et al., 2017). Metal concentrations were determined in the Manchester Royal Infirmary Clinical Biochemistry Department using an Agilent 7700x ICP-MS spectrometer equipped with a MicroMist nebulizer (Glass Expansion, Melbourne, VIC, Australia), a Scott double-post spray chamber and nickel sample and skimmer cones. Samples were introduced into the spray chamber using an Agilent integrated autosampler (I-AS). Before each analysis, the peristaltic-pump sample tubing was replaced to limit impaired sample delivery to the nebulizer. ICP-MS system optimization and performance reports were generated on Agilent MassHunter Workstation software (G7201A, A.01.01) prior to each analysis to ensure consistent system performance.

Scandium was used as the internal standard for all elements except Zn and Se, where germanium was used, and molybdenum (Mo), where indium was used. To remove spectral interferences, two collision-cell gas modes were employed. All elements were analyzed in helium mode (5.0 mL min^–1^ He), except for Se which was analyzed in high-energy helium mode (HEHe; 10 mL min^–1^ He) to reduce interference by polyatomic ion formation. Germanium and indium internal standards were analyzed in both modes. Integration times for relevant trace metals were 3 s for Se; 0.01 s for Fe; 0.03 s for Mn, Cu, and Zn; and 0.1 s for Na, Mg, K, and Ca. A multi-element method using serial dilutions of environmental calibration standards (Supplementary Table 4; Agilent 5183-4688) was implemented for each analytical batch. 50-μg/L and 5-μg/L internal standard calibration solutions were used as periodic quality controls 1 and 2, respectively. The limit of quantification, detection limit, and background equivalent concentrations for each trace-metal analyzed in this report were automatically generated by Agilent MassHunter software (data not shown).

### Data analysis

Wet- and dry-weight raw ICP-MS datasets were first exported to individual Microsoft Excel worksheets where they were corrected for sample weight and dilution and then converted to units of mmol/kg or μmol/kg as appropriate. The means (SD; parametric data) or medians (interquartile range; non-parametric data) were calculated and the significance of inter-group differences were determined by either Welch’s *t*-tests or Mann–Whitney *U* tests based on whether the data were found to be normally distributed by using the Shapiro–Wilks test of normality.

Statistical calculations were performed using GraphPad Prism v8.1.1 (GraphPad; La Jolla, CA, United States). *p*-values < 0.05 were considered significant. *Post hoc* statistical power and sample-size estimates for both dry- and wet-weight brain tissue were calculated using G*Power v3.1.9.4 (Faul et al., 2007).

Receiver operator characteristic (ROC) curves were generated using in GraphPad Prism v8.1.1 (GraphPad; La Jolla, CA, United States) using Na data from all regions that displayed statistically significant case-control differences.

To identify potential metallomic cluster separation between VaD and our previous AD metallomic datasets (Xu et al., 2017), principal component analysis (PCA) was performed using the R-platform MetaboAnalyst (Chong et al., 2019). Metallomic data from the CG, MTG, and HP were used for PCA, as these were the regions that were also present in our prior AD dataset. To assess the suitability of each dataset for PCA, the Kaiser–Meyer–Olkin measure for sampling accuracy and Bartlett’s test of adequacy were first performed using SPSS version 23 (IBM; Armonk, NY, United States). All metal datasets used for PCA were mean-centered and scaled to correct for the use of different units of measurement.

## Results

### Case characteristics

Tissues were obtained from seven regions from each of the 10 brains with cerebrovascular disease and 10 age-/sex-matched controls. No significant differences were detected for age, *post-mortem* delay (PMD), brain weight, or dry-weight/wet-weight tissue ratios (Table 1).

The cause of death for both VaD and control subject cases are reported in Supplementary Tables 1, 2. The cause of death for five cases (32, 92, 131, 170, 347) was not recorded in the SWDBB database. It should also be noted that due to tissue shortages, specimens could not be obtained from the same VaD cohort that was used for our previous investigation of VaD hippocampal tissue (Supplementary Table 1; Philbert et al., 2022). Therefore, these cohort differences will need to be considered when comparing the concentrations of elements.

Despite the considerable efforts made to eliminate all other possible causes of dementia amongst the hippocampal VaD group, the limited availability of hippocampal tissue precluded complete fulfillment of the specified exclusion criteria. In particular, one VaD case (case 1008) in the hippocampal cohort was diagnosed with T2D, which is suggested to independently influence the progression of dementia/cognitive impairment (Xue et al., 2019). However, case 1008 did not appear to generate any detectable outlying values in either dry- or wet-weight metal datasets (Philbert et al., 2022).

### Multiregional elemental differences in vascular dementia brain tissue

Inductively coupled plasma-mass spectrometry analysis was used to measure the concentrations of eight essential metals: Na, Mg, K, Ca, Mn, Fe, Cu, Zn, and the metalloid, Se. Our previous hippocampal metal dataset (Philbert et al., 2022), generated using the same methodology, was also included to allow a more complete overview of the regional brain-metal homeostasis in VaD.

To first establish whether or not any global case-control patterns were apparent in the aforementioned brain-metal datasets, all regions used in the present study were combined and investigated as a single multiregional dataset (VaD, *n* = 70; Con, *n* = 70). Wet-weight global elemental analysis revealed increased Na (*p* < 0.0001) and decreased K levels (*p* = 0.0145) in VaD cases compared to controls (Table 2). However, corresponding dry-weight analysis identified increased Na (*p* = 0.0016) and Se (*p* = 0.0149), as well increased Cu levels (*p* = 0.0008) in VaD cases (Table 3 and Supplementary Figure 1).

**TABLE 2:** Wet weight grand-mean analysis.

Element	Control	VaD	*P*-value
Na	73 (61–83)	91 (80–98)	**<0.0001**
Mg	4.71 (4.31–5.37)	4.66 (4.31–5.14)	0.4935
K	64 (57–76)	59 (53–70)	**0.0145**
Ca	1.53 (1.35–2.02)	1.64 (1.44–2.30)	0.1874
Mn	4.28 (3.40–6.85)	4.10 (3.49–5.28)	0.6217
Fe	0.78 (0.66–1.32)	0.81 (0.65–1.08)	0.8754
Cu	55 (63–71)	64 (47–80)	0.0643
Zn	203 (186–226)	199 (183–225)	0.7031
Se	1.87 (1.61–2.23)	1.95 (1.73–2.32)	0.1099

Data are medians (interquartile range); p-values for significance of between-group differences were calculated by Mann–Whitney U test based on wet-weight measurements from all regions analyzed in this study from control (n = 70) and VaD (n = 70) regional samples. Significant values (p < 0.05) are shown in bold.

**TABLE 3:** Dry weight grand-mean analysis.

Element	Control	VaD	*P*-value
Na	410 (314–507)	494 (384–596)	**0.0016**
Mg	26 (24–28)	26 (23–29)	0.7131
K	340 (307–399)	321 (289–387)	0.0994
Ca	8.87 (7.07–11.56)	8.87 (6.84–12.57)	0.8055
Mn	24 (19–30)	22 (19–30)	0.9652
Fe	4.66 (3.81–5.68)	4.73 (3.77–5.77)	0.7131
Cu	298 (247–358)	353 (279–437)	**0.0008**
Zn	1126 (989–1246)	1183 (936–1326)	0.3112
Se	10.65 (9.74–12.32)	11.50 (10.06–13.11)	**0.0149**

Data are medians (interquartile range); p-values for significance of between-group differences were calculated by Mann–Whitney U test based on dry-weight measurements from all regions analyzed in this study from control (n = 70) and VaD (n = 70) regional samples. Significant values (p < 0.05) are shown in bold.

Given the significance of global Na and Cu concentrations in VaD, linear regression analysis was used to ascertain whether Na levels significantly predicted Cu levels in VaD brain tissue. The fitted regression model for wet- and dry-weight data was *Y* = −0.4696 × X + 105.9 and *Y* = 0.1591 × X + 282.0, respectively. Both regressions were statistically significant (wet-weight *p*-value = 0.0004; dry weight *p*-value = 0.0253); however, the wet-weight regression model identified a negative relationship between global Na and Cu, whereas a positive relationship was observed for the dry-weight data (Supplementary Figure 2).

Global cerebral power analysis was >90% for wet- and dry-weight Na and dry-weight Cu. All other global cerebral metal analyses were <80% (Supplementary Table 15).

### Regional elemental differences in vascular dementia brain tissue

**FIGURE 1:**
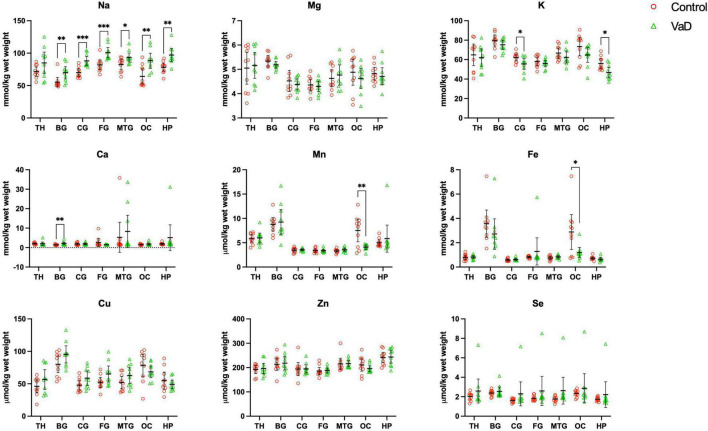
Wet-weight concentrations of nine essential metals **(A–I)** in seven brain regions compared between control (red) and VaD (green) *post-mortem* tissue. Data are means ± 95% CI. TH, thalamus; BG, basal ganglia; CG, cingulate gyrus; FG, frontal gyrus; MTG, middle temporal gyrus; OC, occipital cortex; HP, hippocampus. *p < 0.05, **p < 0.01, and ***p < 0.001.

**FIGURE 2:**
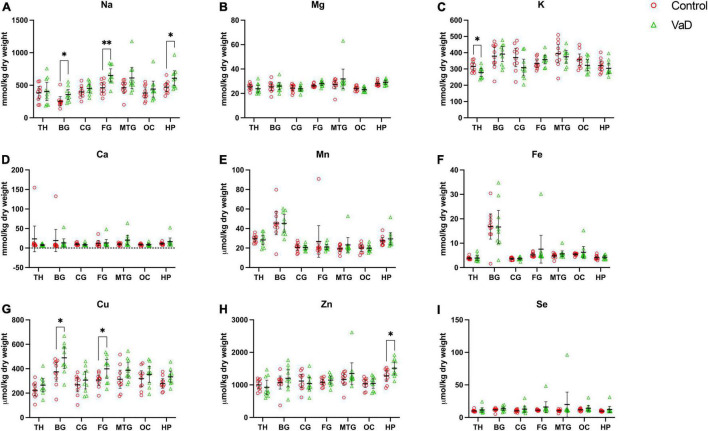
Dry-weight concentrations of nine essential metals **(A–I)** in seven brain regions compared between control (red) and VaD (green) *post-mortem* tissue. Data are means ± 95% CI. TH, thalamus; BG, basal ganglia; CG, cingulate gyrus; FG, frontal gyrus; MTG, middle temporal gyrus; OC, occipital cortex; HP, hippocampus. *p < 0.05, **p < 0.01, and ***p < 0.001.

In regional wet-weight elemental analysis, the most striking case-control difference observed here was the widespread elevation of Na, present in six out of the seven regions analyzed. Markedly increased Na levels were present in the BG (*p* = 0.0089), CG (*p* = 0.0003), FG (*p* = 0.0009), MTG (*p* = 0.0280), OC (*p* = 0.0038), and HP (*p* = 0.0038) [Figure 1 and Tables 4–9; see Philbert et al. (2022) for hippocampal metal levels]. Although mean Na levels in the TH trended higher in the VaD group compared to controls, these differences were not statistically significant (Figure 1 and Table 5). Wet-weight Na concentrations were highest in the FG (100 mmol/kg) and lowest in BG (70 mmol/kg); however, the OC showed the greatest fold-change (fc) increase (1.38 fc) and the MTG the lowest (1.13 fc). Such Na perturbations were less apparent in dry-weight analysis, as only the BG (*p* = 0.038), FG (*p* = 0.0052), and HP (*p* = 0.0326) presented increased Na levels in VaD tissue (Figure 2 and Supplementary Tables 10, 12, 14, respectively). While changes in the other regions were not statistically significant (TH, CG, MTG, OC), mean Na levels in dry-weight analyses for these regions all trended higher in the VaD group compared to controls. This may reflect lower sensitivity in dry-weight analyses.

**TABLE 4:** Wet-weight metal concentrations in the middle temporal gyrus of VaD and control brains.

Element	Units	Reference isotope	Control	VaD	*P*-value
Na	mmol/kg	^23^Na	83 (11)	93 (10)	**0.0280**
Mg	mmol/kg	^23^Mg	4.63 (0.48)	4.77 (0.58)	0.5713
K	mmol/kg	^39^K	67 (7)	62 (8)	0.2178
Ca	mmol/kg	^44^Ca	5.28 (10.76)	8.30 (11.57)	0.5530
Mn	μmol/kg	^55^Mn	3.30 (0.40)	3.54 (0.46)	0.2219
Fe	mmol/kg	^56^Fe	0.74 (0.18)	0.84 (0.15)	0.1994
Cu	μmol/kg	^63^Cu	52 (52)	63 (63)	0.1311
Zn	μmol/kg	^66^Zn	215 (32)	216 (17)	0.9315
Se	μmol/kg	^78^Se	1.76 (0.29)	2.63 (1.93)	0.1925

Data are means (SD); p-values for significance of between-group differences were calculated by Welch’s t-test based on wet-weight measurements from control (n = 10) and VaD (n = 10) brains. Significant values (p < 0.05) are shown in bold.

**TABLE 5:** Wet-weight metal concentrations in the thalamus of VaD and control brains.

Element	Units	Reference isotope	Control	VaD	*P*-value
Na	mmol/kg	^23^Na	72 (10)	85 (23)	0.1201
Mg	mmol/kg	^23^Mg	5.05 (0.92)	5.16 (0.74)	0.7704
K	mmol/kg	^39^K	65 (16)	62 (13)	0.6458
Ca	mmol/kg	^44^Ca	2.02 (0.49)	1.99 (1.16)	0.9393
Mn	μmol/kg	^55^Mn	5.85 (1.11)	5.98 (1.40)	0.8312
Fe	mmol/kg	^56^Fe	0.80 (0.25)	0.82 (0.17)	0.8437
Cu	μmol/kg	^63^Cu	46 (14)	57 (21)	0.2049
Zn	μmol/kg	^66^Zn	192 (23)	196 (31)	0.7680
Se	μmol/kg	^78^Se	2.01 (0.41)	2.59 (1.72)	0.3292

Data are means (SD); p-values for significance of between-group differences were calculated by Welch’s t-test based on wet-weight measurements from control (n = 10) and VaD (n = 10) brains.

**TABLE 6:** Wet-weight metal concentrations in the basal ganglia of VaD and control brains.

Element	Units	Reference isotope	Control	VaD	*P*-value
Na	mmol/kg	^23^Na	52 (51–55)	71 (58–78)	**0.0089**
Mg	mmol/kg	^23^Mg	5.37 (5.12–5.77)	5.17 (5.06–5.31)	0.2176
K	mmol/kg	^39^K	80 (75–85)	76 (70–81)	0.2176
Ca	mmol/kg	^44^Ca	1.36 (1.25–1.50)	1.83 (1.50–2.43)	**0.0039**
Mn	μmol/kg	^55^Mn	8.90 (7.33–9.67)	7.55 (7.55–11.91)	0.9118
Fe	mmol/kg	^56^Fe	3.46 (2.57–3.86)	2.45 (1.70–3.05)	**0.0630**
Cu	μmol/kg	^63^Cu	84 (60–96)	96 (79–102)	0.1051
Zn	μmol/kg	^66^Zn	208 (197–238)	207 (188–249)	>0.9999
Se	μmol/kg	^78^Se	2.40 (2.14–2.59)	2.30 (2.11–2.86)	0.7959

Data are medians (interquartile range); p-values for significance of between-group differences were calculated by Mann–Whitney U test based on wet-weight measurements from control (n = 10) and VaD (n = 10) brains. Significant values (p < 0.05) are shown in bold.

**TABLE 7:** Wet-weight metal concentrations in the occipital cortex of VaD and control brains.

Element	Units	Reference isotope	Control	VaD	*P*-value
Na	mmol/kg	^23^Na	64 (16)	88 (16)	**0.0038**
Mg	mmol/kg	^23^Mg	4.88 (0.75)	4.62 (0.57)	0.3929
K	mmol/kg	^39^K	74 (13)	65 (11)	0.1284
Ca	mmol/kg	^44^Ca	1.53 (0.31)	1.80 (0.79)	0.3340
Mn	μmol/kg	^55^Mn	7.51 (3.23)	4.08 (0.61)	**0.0085**
Fe	mmol/kg	^56^Fe	2.88 (2.01)	1.19 (0.59)	**0.0277**
Cu	μmol/kg	^63^Cu	78 (24)	68 (13)	0.2832
Zn	μmol/kg	^66^Zn	211 (36)	195 (17)	0.2459
Se	μmol/kg	^78^Se	2.33 (0.11)	2.88 (0.66)	0.4398

Data are means (SD); p-values for significance of between-group differences were calculated by Welch’s t-test based on wet-weight measurements from control (n = 10) and VaD (n = 10) brains. Significant values (p < 0.05) are shown in bold.

**TABLE 8:** Wet-weight metal concentrations in the frontal gyrus of VaD and control brains.

Element	Units	Reference isotope	Control	VaD	*P*-value
Na	mmol/kg	^23^Na	82 (10)	101 (11)	**0.0009**
Mg	mmol/kg	^23^Mg	4.36 (0.34)	4.30 (0.30)	0.6880
K	mmol/kg	^39^K	58 (6)	56 (5)	0.3887
Ca	mmol/kg	^44^Ca	2.61 (2.68)	1.50 (0.19)	0.2228
Mn	μmol/kg	^55^Mn	3.37 (0.48)	3.37 (0.37)	0.9913
Fe	mmol/kg	^56^Fe	0.82 (0.10)	1.28 (1.56)	0.3697
Cu	μmol/kg	^63^Cu	52 (11)	65 (17)	0.0654
Zn	μmol/kg	^66^Zn	185 (21)	188 (14)	0.7395
Se	μmol/kg	^78^Se	1.84 (0.28)	2.59 (2.10)	0.2882

Data are means (SD); p-values for significance of between-group differences were calculated by Welch’s t-test based on wet-weight measurements from control (n = 10) and VaD (n = 10) brains. Significant values (p < 0.05) are shown in bold.

**TABLE 9:** Wet-weight metal concentrations in the cingulate gyrus of VaD and control brains.

Element	Units	Reference isotope	Control	VaD	*P*-value
Na	mmol/kg	^23^Na	70 (8)	88 (10)	**0.0003**
Mg	mmol/kg	^23^Mg	4.52 (0.61)	4.38 (0.32)	0.5193
K	mmol/kg	^39^K	62 (5)	56 (8)	**0.0338**
Ca	mmol/kg	^44^Ca	1.85 (0.68)	1.89 (0.66)	0.8826
Mn	μmol/kg	^55^Mn	3.46 (0.50)	3.55 (0.29)	0.6432
Fe	mmol/kg	^56^Fe	0.59 (0.59)	0.62 (0.12)	0.4918
Cu	μmol/kg	^63^Cu	48 (10)	59 (16)	**0.0834**
Zn	μmol/kg	^66^Zn	194 (37)	195 (23)	0.9823
Se	μmol/kg	^78^Se	1.61 (0.21)	2.30 (1.73)	0.2428

Data are means (SD); p-values for significance of between-group differences were calculated by Welch’s t-test based on wet-weight measurements from control (n = 10) and VaD (n = 10) brains. Significant values (p < 0.05) are shown in bold.

Analysis of cerebral K concentrations also revealed perturbations in several regions, although, these differences were not as widespread as for Na. Analysis of wet-weight tissue revealed evidence for decreased K in the CG (*p* = 0.0338) and HP (*p* = 0.0101). For dry-weight analysis, decreased K was only found in the TH (*p* = 0.0237) (Figure 2 and Supplementary Table 9). However, in contrast to wet tissue, decreases in K in the VaD group were apparent in fewer regions in dry-weight analysis; while not statistically significant, mean K levels trended higher in the BG and FG compared to controls. Na and K were the only two metals that were consistently perturbed in both dry- and wet-weight analyses.

In addition, wet-weight analysis revealed moderate evidence for increased Ca in the BG (*p* = 0.0039), as well as decreased Mn in the OC (*p* = 0.0085) and Fe in the OC (*p* = 0.0277).

Interestingly, dry-weight analysis provided some evidence for Cu and Zn perturbations that were not evident in the corresponding wet-weight analysis. In VaD, Cu was significantly increased in both BG (*p* = 0.0370) and FG (*p* = 0.0317) dry tissue. A non-significant trend toward elevated Cu levels was also detected in wet-weight VaD tissue in these regions. Dry-weight Zn levels were higher in VaD tissue than controls in the hippocampus (*p* = 0.0447), however, no trend was evident in the equivalent wet-weight analysis. No other physiological metals from either wet- or dry-weight analyses in the present study showed any significant case-control differences.

Of the metals that were perturbed, when separated by gender (males = 4; females = 6), mean metal levels showed mixed results for both sets of analyses. However, in wet-weight analysis, female VaD brain tissue displayed higher mean-Na levels compared to male VaD tissue for all brain regions.

**FIGURE 3:**
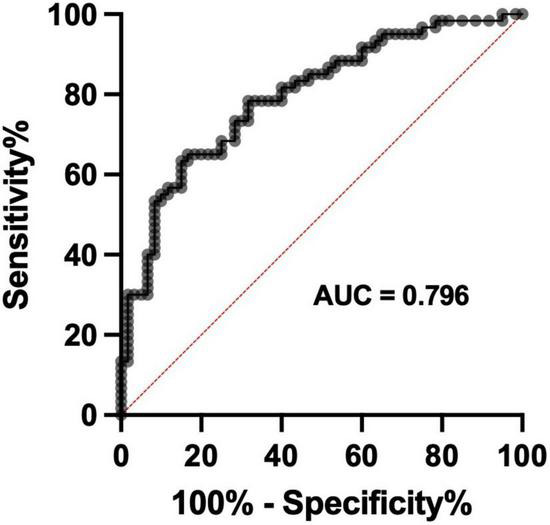
Receiver operator characteristic curve for wet-weight cerebral Na levels. Data represents wet-weight cerebral-Na measurement in all regions that displayed significant case-control Na perturbations. *n* = 120 (Con = 60; VaD = 60).

Receiver operator characteristic curve analysis was employed to determine the diagnostic potential of cerebral-Na levels in VaD. Only wet-weight data were used for this ROC curve analysis as it provides a better reflection of the actual physiological metal levels in brain tissue. In addition, the CV values were marginally lower in the wet-weight tissue when compared to the dry-weight tissue, which provided a further rationale for the inclusion of only wet-weight data for ROC curve analysis (Supplementary Tables 5–7). Wet-weight cerebral-Na data from all regions that displayed statistically significant case-control differences were compiled into a single spreadsheet and used for ROC curve analysis [*n* = 120 (Con = 60, VaD = 60)]. The ROC curve displayed an area under the curve (AUC) of 0.796 (*p* < 0.0001; 95% CI = 0.717–0.875), consistent with fair discriminatory power for cerebral-Na levels in classifying between VaD and controls (Figure 3).

Due to the limited sample numbers in the present study, *post-hoc* power tests and sample-size estimates were conducted to ensure the reliability of the observed case-control differences. Wet-weight power levels were >80% and minimum sample sizes were <20 for Na in four regions, as well as for Ca in the BG and Mn in the OC. For dry-weight analyses, Na in the FG was the only metal to satisfy the statistical power and sample-estimate criteria (Supplementary Tables 16, 17).

### Elemental comparisons with Alzheimer’s disease

The VaD metal concentrations reported here were then compared with our group’s previous analysis of AD *post-mortem* brain tissue, generated using the same ICP-MS methodology (Xu et al., 2017). Seven regions from AD brains were investigated by Xu et al. (2017), including the HP, entorhinal cortex (ENT), MTG, sensory cortex (SCX), motor cortex (MCX), CG, and cerebellum (CB); however, only three regions (HP, MTG, and CG) overlapped with those analyzed in the present study of VaD. Therefore, direct comparisons between VaD and AD could only be made for these three regions. Interestingly, both the HP and MTG in AD exhibited similar Na elevations as VaD wet-weight tissue in these regions. However, unlike VaD, elevated Na levels were only seen in the most severely affected regions in AD (HP, ENT, and MTG). Furthermore, K was also shown to be decreased in AD CG tissue, but not the HP or MTG.

Perhaps the most striking observation from Xu et al. (2017) was the widespread decrease in Cu in all regions. In contrast, here, dry-weight analysis of the BG and FG, as well as global case-control analysis was consistent with increased Cu levels in VaD (Supplementary Tables 10, 12, Supplementary Figure 1, and Table 3).

While several other AD-related perturbations were observed in these regions, including Zn, Fe, Mn, Se, and Mg, these perturbations were not seen in the same regions in VaD brain tissue.

To further understand the combined metallomic differences identified in our VaD and AD datasets, PCA was conducted to assess the overall metallomic pattern of these disease states. As previously explained, only the HP, MTG, and CG datasets were used for PCA due to the overlap of metallomic data between AD and VaD datasets. Where applicable, if control groups from both VaD and AD cohorts displayed substantial overlap, then they were grouped and used as a single control cohort against all cases thereafter (as planned *a priori*). In the dry-weight MTG PCA dataset, one sample from the AD group and two from the AD controls were removed as a result of missing values to enable dimension reduction. No other samples from either the HP or CG PCA dry-weight datasets were excluded. However, several samples in the wet-weight investigation of AD were unable to be analyzed due to the limited availability of tissue.

Prior to PCA, the suitability of the analytical methods was assessed. The Kaiser–Meyer–Olkin (KMO) measure for sampling accuracy was 0.598 for MTG, 0.690 for CG, and 0.580 for HP dry-weight datasets. For wet-weight analysis, the KMO was 0.512 for MTG, 0.669 for CG, and 0.695 for HP datasets. As there is some debate regarding whether a KMO of 0.5 or 0.6 should be used as the minimum threshold for dimension reduction, all three regions from both dry- and wet-weight datasets were used for PCA. However, these KMO scores are described as being mediocre, which should be taken into account when interpreting these data (Kaiser and Rice, 1974). Bartlett’s test of sphericity was statistically significant for all three regions from both tissue conditions (*p* < 0.0001), which provides strong evidence for significant correlation between variables to support data reduction. PCA revealed no separation between all cases and controls used for both dry- (Supplementary Figure 3) and wet-weight data (Supplementary Figure 4), which implies that there are no differences in the overall metallomic profile between AD and VaD for these regions.

### Widespread Na/K ratio increases in vascular dementia tissue

In our previous pilot study (Philbert et al., 2022), increased Na/K ratios (consistent with high Na and low K levels) were identified in VaD cases. Hence, Na/K ratios were calculated from all regional metal datasets generated here from both dry- and wet-weight analyses to determine whether the same hippocampal increases were found globally or were specific to certain brain regions. Global analysis of cerebral Na/K ratios identified increased VaD Na/K ratios compared to controls in both wet- (*p* = < 0.0001) and dry-weight analysis (*p* = 0.0001) (Supplementary Table 18). When separated by region, VaD Na/K ratio increases were found in the BG [wet weight (*p* = 0.0039); dry weight (*p* = 0.0106)], CG [wet weight (*p* = 0.0003); dry weight (*p* = 0.0012)], FG [wet weight (*p* = 0.0089); dry weight (*p* = 0.0157)], and HP [wet weight (*p* = 0.0039); dry weight (*p* = 0.0314)] (Supplementary Tables 19, 20 and Supplementary Figure 5). When these ratios were compared with overlapping regions from our prior AD dry-weight metal dataset (CG, MTG, HP) (Xu et al., 2017), the MTG (*p* = 0.0011) and HP (*p* = 0.0006), but not CG, showed similar Na/K ratio increases (Supplementary Table 21). However, in regions where Na/K ratio perturbations were evident, the Na/K ratio fc was higher in AD compared to both wet and dry VaD *post-mortem* brain tissue.

## Discussion

Numerous studies have provided evidence for brain-metal dyshomeostasis in several neurodegenerative conditions (Xu et al., 2017; Violi et al., 2020; Scholefield et al., 2021). In contrast, the molecular events that drive cerebrovascular pathology and lead to VaD are obscure. Our recent data describing metal levels in the hippocampus of subjects with VaD (Philbert et al., 2022) suggested that metallomic perturbations may play a pivotal role in neurodegeneration in VaD. To our knowledge, a complete multiregional metallomic investigation using sensitive mass-spectrometry techniques has yet to be reported for human *post-mortem* brains with small vessel disease related dementia.

Here we report the concentrations of eight essential metals and Se in seven brain regions from 10 VaD cases and 10 age-/sex-matched controls. No differences were observed for age, sex, *post-mortem* delay (PMD), brain weight, or wet-weight/dry-weight ratio between VaD cases and controls in the present study. This indicates that such variables are unlikely to have affected the brain-metal differences seen in this study. It is also worth noting that while the PMDs for the cohorts used in this study were not unusually high for United Kingdom-based brain banks, it has been suggested that extended PMDs can alter analyte expression. However, based on our group’s previous findings, it is unlikely that the PMDs presented in this study significantly altered brain-metal levels (Scholefield et al., 2020).

The precise maintenance of transmembrane Na^+^ fluxes and intracellular Na^+^ homeostasis is fundamental to the propagation of action potentials and the movement of ions across the cell membrane (Coppini et al., 2013). Here, widespread wet-weight Na elevations in VaD cases were observed in six out of the seven brain regions analyzed. These data mirror our group’s previous study, wherein increased Na levels were observed in the hippocampus of VaD brains (which values have been included in the present study for comparison) (Philbert et al., 2022). Despite limitations in our methodology which prevent the specific detection of intra- or extracellular ions, prior reports of Na^+^ and K^+^ ion perturbations in AD brain tissue without cation differences in corresponding cerebrospinal fluid may imply that such ion imbalances are a reflection of intracellular differences rather than changes in the extracellular compartment (Vitvitsky et al., 2012). It is also certainly plausible that systemic Na could have crossed the blood-brain barrier into the CNS and affected the cerebral Na measurements shown here. However, elevated systemic Na levels (hypernatremia) are not known to occur in VaD patients and were not reported in the VaD brain-bank metadata for these cases. Furthermore, although hypernatremia is more frequent in elderly individuals, it primarily manifests due to dehydration (Adrogue and Madias, 2000). Yet here, no differences in brain-water content between cases and controls were observed. Therefore, it is unlikely that the cerebral Na perturbations shown here are a product of hypernatremia. To the authors’ knowledge, global cerebral elevations of Na have not previously been reported in VaD patients, hence these data represent a novel mechanistic insight into the pathophysiology of VaD. Furthermore, in our previous multiregional investigation of AD (Xu et al., 2017), increased cerebral Na levels were only observed in severely affected regions. Thus, the presence of widespread Na elevation in the VaD brain could potentially be used to assist in the differential diagnosis of AD and VaD.

To assess the diagnostic potential of Na measurement in *post-mortem* VaD brain tissue, ROC curves using Na ICP-MS data were generated from brain regions that displayed significant case-control differences. A ROC curve with an area under the curve of 0.796 was identified which demonstrated fair discriminatory power for cerebral-Na levels in classifying between VaD and controls. These data indicate the possible application of clinical ^23^Na magnetic resonance imaging in the diagnosis of VaD, which has been previously validated in multiple sclerosis patients (Inglese et al., 2010; Eisele et al., 2016; Weber et al., 2021).

Due to the high metabolic costs of neuronal computation, the central nervous system (CNS) uses ∼20% of the body’s resting ATP production via Na^+^/K^+^-ATPase to mediate regulation of intracellular Na^+^ and K^+^ concentrations (Harris et al., 2012). Previously (Philbert et al., 2022), we proposed the idea that contrasting perturbations of Na and K in the hippocampus of VaD brains may be due to the dysfunction of the Na^+^/K^+^-ATPase. Given the observation of widespread Na elevations in VaD brains here, it is probable that the proposed Na^+^/K^+^-ATPase dysfunction is a global molecular event in VaD. Reduced cerebral-glucose metabolism (hypometabolism) has been documented in both AD and VaD cases, which is crucial to the initial stages of cellular respiration and the subsequent generation of ATP (Kerrouche et al., 2006; Seo et al., 2009; Sanabria-Diaz et al., 2013). This gives reason to believe that defective energy utilization leading to diminished pools of ATP could lead to the observed intracellular Na elevations seen here that are likely due to reduced Na^+^/K^+^-ATPase activity. This is further supported by proteomic evidence showing downregulation of ATP synthase (electron transport chain complex V; EC 7.1.2.2) subunits (Datta et al., 2014; Wang et al., 2015) and upregulation of Na/K-ATPase alpha subunit 3 (Adav et al., 2014) in VaD middle-temporal gyrus *post-mortem* tissue. Reduced activity of Na/K-ATPase can also lead to neuronal cell death (Xiao et al., 2002; Kurauchi et al., 2018) potentially via glutamate neurotoxicity and alterations in dopamine release (Lees, 1991), which could further exacerbate cerebrovascular pathology in VaD. However, measurement of Na/K-ATPase activity and abundance will be necessary to fully confirm its dysregulation in VaD.

Interestingly, when compared with our prior hippocampal metal dataset (Philbert et al., 2022), only the cingulate gyrus exhibited similar wet-weight K level decreases in VaD cases. However, in wet- but not dry-weight analysis, global K levels were lower in VaD cases than controls. This gives reason to believe that there may be other mechanisms that contribute to the Na elevation in VaD such as altered function of voltage-gated Na channels (Carter, 1998), the Na^+^/Ca^+^ exchanger (Shenoda, 2015), or the Na^+^/H^+^ exchanger (Bobulescu et al., 2005). However, the extent of their contribution here requires further clarification, particularly in the case of the Na^+^/Ca^+^ exchanger as Ca was shown to only be increased in wet-weight VaD basal ganglia tissue. A further possible reason for the lack of hypothesized regional cerebral-K depletion in these results may be due to the pump dynamics of the Na^+^/K^+^-ATPase. Through the hydrolysis of one ATP molecule, three Na^+^ ions are pumped out of the cell and two K^+^ are pumped into the cell (Lingrel and Kuntzweiler, 1994). Therefore, during periods of reduced pump activity, perturbations in Na may appear greater than in K and hence the lack of statistical difference may merely reflect low sample numbers rather than actual intergroup K differences. The need for a larger sample size is further reinforced by the lower statistical power (<80%) for K observed in both wet- and dry-weight tissue for all regions.

In addition to controlling intracellular ionic gradients, the Na^+^/K^+^-ATPase also plays a crucial role in maintaining osmotic equilibrium within the cell (Pivovarov et al., 2018). In the present study, no case-control differences were observed for water percentage or total-brain weight for all brain regions analyzed (Table 1), which indicates the absence of cerebral edema. However, previous reports of brain-water content in the presence of opposing intracellular Na and K concentration gradients have reportedly shown tissue water-content elevation (Young et al., 1987; Yang et al., 1992). Although these reports may differ from those in the present study, the aforementioned studies relate to the acute response after Na^+^/K^+^-ATPase disruption, whereas, here, dysfunction of the Na^+^/K^+^-ATPase in these VaD tissues almost certainly reflects chronic events. Therefore, given the understanding that VaD patients may be subject to chronic Na^+^/K^+^-ATPase dysfunction, the absence of elevated water percentage or brain weight in the VaD group might be presumed to reflect the further involvement of other Na^+^ exchangers and/or compensatory mechanisms. However, what these compensatory mechanisms are will require further clarification.

To augment our understanding of Na^+^/K^+^-ATPase dysfunction in VaD brain tissue, Na/K ratios (consistent with high Na and low K levels) were calculated. Several reports have also highlighted the utility of high Na/K ratios as a risk factor in cardiovascular disease and stroke (Averill et al., 2019; Jung et al., 2019), consistent with its application here. Na/K ratios were increased in four regions in both wet- and dry-weight VaD tissue, with most of the remaining regions trending toward increased ratios. In addition, global analysis of the cerebral Na/K ratio identified increased VaD ratios in both wet- and dry-tissue datasets. This suggests that despite the absence of measurable regional cerebral-K decreases, the observed global and regional Na/K ratios highlight a profound disturbance of cerebral Na^+^ and K^+^ homeostasis in VaD.

The study of physiological Cu levels has gained considerable attention in recent years, not only because of its essentiality in key enzymatic reactions (Uauy et al., 1998), but also due to the likely role that Cu plays in neurodegenerative disease (Waggoner et al., 1999). Previous reports have described widespread cerebral copper deficiency in AD (Xu et al., 2017) and PDD (Scholefield et al., 2021) to levels that might exacerbate neurodegenerative pathology. Although VaD is known to manifest concomitant AD-type neurodegenerative pathology (Kalaria, 2002), here only dry-weight VaD tissue from the BG and FG displayed increased Cu levels. No other statistically significant differences were detected for Cu in the remaining regions for either wet- or dry-weight analyses. However, global cerebral elemental comparisons identified substantive evidence for increased dry-weight Cu levels in VaD cases. The contrasting global Cu increases in VaD compared with the widespread lowering of levels previously reported in AD (Xu et al., 2017) and PDD (Scholefield et al., 2021), therefore indicates that the VaD metallomic profile is fundamentally different from these other age-related dementias. Thus, these current data may imply that elevated brain Cu in VaD may predominantly be driven by cerebrovascular pathology. Although, despite observed Cu differences in the BG and FG, only global dry-weight brain-Cu levels satisfied our requirement for *post hoc* statistical power (<80%) (Supplementary Tables 15, 17). Therefore, the lack of statistical power in the BG and FG must be considered when interpreting these results.

The finding of increased global brain-Cu levels in VaD is consistent with previous reports of elevated serum-Cu levels in VaD (Agarwal et al., 2008), as well as an increased risk of ischemic stroke (Xiao et al., 2019). While research surrounding cerebral-Cu homeostasis in VaD is lacking, the effects of chronic cerebral-Cu toxicity have been firmly established in Wilson’s disease, an autosomal recessive disorder which causes neurodegeneration due to excess Cu levels (Das and Ray, 2006). Although the Cu elevation seen here is not equivalent to that of Wilson’s disease (Cumings, 1948), elevated-brain copper nonetheless has the potential to contribute to the pathogenesis of VaD, presumably through the formation of reactive oxygen species and increased neuroinflammation (Valko et al., 2016).

Although multiregional cerebral-Na elevations in VaD remains the focal point of this investigation, concomitant global cerebral-Cu levels are of particular interest given that both metals are tightly associated with bioenergetics. Therefore, to further understand the relevance of these perturbations in relation to one another, linear regression analysis was employed to determine whether Na levels predicted Cu levels in VaD brain tissue. Interestingly, the regression models identified both negative and positive relationships between Na and Cu in wet- and dry-weight tissue, respectively. Although the levels of VaD Na and Cu in the present dataset are of critical importance, further analysis will be needed to fully determine why these differences between wet- and dry-weight regressions exist, given that these data were generated from the same samples.

Perturbations in single regions were also observed for Ca, Mn, and Fe in wet-weight analysis, and Zn in dry-weight analysis. Although both Ca and Mn satisfied our criteria for statistical power, these differences were not present in both dry- and wet-weight analysis. Therefore, further investigation will be required to fully understand the relevance of these findings.

As both VaD and AD present clinical and pathological similarities (Kalaria, 2002), the multiregional VaD metal data here were combined with our previous AD dataset (Xu et al., 2017), generated using the same methodology, to further understand the metallomic relationship between these two diseases. Due to differences in the regions sampled between the two studies, only three regions (HP, MTG, and CG) contained metallomic data from both AD and VaD. No apparent differences in the metallomic pattern were seen amongst the HP, MTG, and CG between VaD and AD using PCA. Contrary to the disease-specific metal perturbations observed in both AD and VaD, this observation further reinforces the reported similarity between these two diseases. However, this similarity only concerns the metallome; therefore, untargeted multi-omic analysis will be necessary to identify possible similarities between VaD and AD in other omics fields.

In the present study, CV values were generally lower in the wet-weight compared to the dry-weight analysis. One possible explanation for the higher CV observed in the dry-weight group may be due to the greater number of analytical steps required. As all dry-weight samples used for ICP-MS analysis underwent an extra day of drying and weighing before measurement, this might have inadvertently increased the variability amongst these samples. Therefore, wet-weight metal measurement may be the preferred method in the present study and for future ICP-MS analyses.

In addition to low statistical power, other limitations in this study also include potential oncological impact, lack of patient medical records, and lack of diagnostic detail. Several controls, but none of the VaD cases, had cancer (as well as other causes not related to cancer) listed in their cause of death. Some chemotherapies can cause decreased systemic Na levels (Ezoe et al., 2018) and chemotherapy is known to cause brain damage which can also alter Na levels (Bradshaw and Smith, 2008). Despite this, no clear distinctions in Na levels were evident between controls with cancer and controls without cancer. Nonetheless, further elemental analysis may be required to confirm the effects of cancer on Na levels in *post-mortem* brain tissue. In addition, patient medical records for example, blood pressure and previous medication were not available for many of the controls and VaD cases used in this study. This information would be of critical importance to the understanding of Na homeostasis in the brain, however, such data is not routinely collected by United Kingdom brain banks which represents an innate flaw in the use of *post-mortem* tissue. Lastly, diagnostic data including cause of death, Braak stage, and CAA were also not specified for several cases. This was due to the limited availability of metadata in the brain bank database using the specified sample inclusion criteria. While *post-mortem* examination confirmed the absence of concomitant neurodegenerative diseases capable of causing dementia (e.g., AD) in all samples, such diagnostic data would have been valuable for further understanding the mechanism(s) responsible for elevated brain-Na levels. Follow-up analysis may be necessary using Braak stage and CAA at different stages to assess their impact on brain-metal levels in VaD.

To the Authors’ knowledge, this is the first study to provide a multiregional assessment of cerebral-metal levels in VaD *post-mortem* tissue using ICP-MS. In conclusion, these results present novel evidence for severe widespread Na elevation in VaD brains. Importantly, this pattern of Na elevation was not apparent in our group’s previous investigation of AD brains, measured using the same methodology. This highlights the potential application of Na as a diagnostic biomarker in VaD patients via clinical ^23^Na magnetic resonance imaging, which may also assist in the *ante-mortem* discrimination of VaD from AD. Furthermore, decreased brain-K levels as well as increased Na/K ratios were also observed in several regions in the VaD group. This implies that the Na^+^/K^+^-ATPase may be functioning at a sub-optimal level, probably due to defective energy utilization, thus contributing to the contrasting Na and K levels seen here. However, it is likely that additional mechanisms such as alterations in voltage-gated Na channels and the Na^+^/H^+^ exchanger are also contributing to the Na and K perturbations seen here. Further studies will be required to confirm the diagnostic utility of clinical brain-Na levels and the precise involvement of the Na^+^/K^+^-ATPase in VaD.

## Data Availability Statement

The original contributions presented in this study are included in the article/Supplementary Material, further inquiries can be directed to the corresponding author.

## Author contributions

SP designed and performed the experiments, analyzed and interpreted the data, and wrote the manuscript. JX performed the experiments and revised the manuscript. SC performed the experiments and analyzed the data. RU designed the experiments, interpreted the data, and revised the manuscript. FR advised on the sampling of brain regions and revised the manuscript. GC designed the experiments, interpreted the data, revised the manuscript, and bears overall responsibility for the integrity of this manuscript and of the study. All authors contributed to the article and approved the submitted version.

## Conflict of Interest

The authors declare that the research was conducted in the absence of any commercial or financial relationships that could be construed as a potential conflict of interest.

## Publisher’s Note

All claims expressed in this article are solely those of the authors and do not necessarily represent those of their affiliated organizations, or those of the publisher, the editors and the reviewers. Any product that may be evaluated in this article, or claim that may be made by its manufacturer, is not guaranteed or endorsed by the publisher.

## Abbreviations

BG: basal ganglia
Ca: calcium
CG: cingulate gyrus
Cu: copper
Fe: iron
FG: frontal gyrus
HP: hippocampus
K: potassium
Mg: magnesium
Mn: manganese
MTG: middle temporal gyrus
Na: sodium
OC: occipital cortex
Se: selenium
TH: thalamus
Zn: zinc.
