# Supplementary material for: Pan-cerebral sodium elevations in vascular dementia: Evidence for disturbed brain-sodium homeostasis
Source: Front Aging Neurosci. 2022 Jul 18;14:926463. doi: 10.3389/fnagi.2022.926463 (PMC9340791; doi:10.3389/fnagi.2022.926463)
Supplement: Supplementary file 1 [file Data_Sheet_1.docx]

Supplementary Material

Supplementary Tables

[Supplementary Table 1. Hippocampal tissue individual patient characteristics 3](#_Toc105156708)

[Supplementary Table 2. SWDBB multiregional individual patient characteristics 4](#_Toc105156709)

[Supplementary Table 3. Group characteristics for the hippocampal cohort 5](#_Toc105156710)

[Supplementary Table 4. ICP-MS calibration standard solutions 5](#_Toc105156711)

[Supplementary Table 5. Coefficient of variations for wet-weight ICP-MS analysis 6](#_Toc105156712)

[Supplementary Table 6. Coefficient of variations for dry-weight ICP-MS analysis 6](#_Toc105156713)

[Supplementary Table 7. Mean coefficient of variations for multiregional wet- and dry-weight ICP-MS analysis 7](#_Toc105156714)

[Supplementary Table 8. Dry-weight metal concentrations in the middle temporal gyrus of VaD and control brains 8](#_Toc105156715)

[Supplementary Table 9. Dry-weight metal concentrations in the thalamus of VaD and control brains 8](#_Toc105156716)

[Supplementary Table 10. Dry-weight metal concentrations in the basal ganglia of VaD and control brains 9](#_Toc105156717)

[Supplementary Table 11. Dry-weight metal concentrations in the occipital cortex of VaD and control brains 9](#_Toc105156718)

[Supplementary Table 12. Dry-weight metal concentrations in the frontal gyrus of VaD and control brains 10](#_Toc105156719)

[Supplementary Table 13. Dry-weight metal concentrations in the cingulate gyrus of VaD and control brains 10](#_Toc105156720)

[Supplementary Table 14. Dry-weight metal concentrations in the hippocampus of VaD and control brains 11](#_Toc105156721)

[Supplementary Table 15. Global brain post-hoc power analysis 12](#_Toc105156722)

[Supplementary Table 16. Multiregional post-hoc power estimates 13](#_Toc105156723)

[Supplementary Table 17. Multiregional sample size estimates 14](#_Toc105156724)

[Supplementary Table 18. Global brain Na/K ratio comparisons between VaD and control brain tissue 15](#_Toc105156725)

[Supplementary Table 19. Wet-weight Na/K ratio comparisons between VaD and control brain tissue 16](#_Toc105156726)

[Supplementary Table 20. Dry-weight Na/K ratio comparisons between VaD and control brain tissue 17](#_Toc105156727)

[Supplementary Table 21. Dry-weight Na/K ratio comparisons between AD and control brain tissue 18](#_Toc105156728)

**Supplementary Figures**

Supplementary Figure 1. Grand-mean Cu analysis from wet- and dry-weight post-mortem brain tissue 19

Supplementary Figure 2. Simple linear regression plots between dry- and wet-weight sodium and copper levels in VaD brain tissue.. 20

Supplementary Figure 3. Two dimensional PCA plots for human dry-weight post-mortem tissue from AD and VaD. 21

Supplementary Figure 4. Two dimensional PCA plots for human wet-weight post-mortem tissue from AD and VaD. 22

Supplementary Figure 5. Individual multiregional Na/K ratios for wet- and dry-weight post-mortem brain tissue.. 23

| Supplementary Table 1. Hippocampal tissue individual patient characteristics | | | | | | | | |
| --- | --- | --- | --- | --- | --- | --- | --- | --- |
| Code | Class | Age at death | Sex | Brain wt (g) | PMD (h) | Braak stage | CAA | Cause of death^*^ |
| 72 | Control | 72 | M | 1300 | 42 | Not specified | Not specified | Ruptured abdominal aortic aneurysm, ischaemic heart disease |
| 122 | Control | 82 | M | 1480 | 30 | Not specified | Not specified | Congestive cardiac failure, ischaemic heart disease, UTI |
| 781 | Control | 87 | M | 1364 | 24 | II | Mild CAA | Acute renal failure, myeloma |
| 930 | Control | 94 | F | 1218 | 29.5 | II | Moderate CAA | Oesophageal carcinoma, ischemic heart disease |
| 943 | Control | 70 | F | 1032 | 33.25 | II | Absent | Carcinomatosis, metastatic squamous cell carcinoma of larynx |
| 948 | Control | 82 | F | 1135 | 36 | II | Mild CAA | Carcinomatosis, ovarian/peritoneal carcinoma |
| 957 | Control | 86 | M | 1345 | 44.25 | II | Mild CAA | Infective exacerbation of COPD |
| 1067 | Control | 69 | F | 1121 | 38.25 | I | Absent | Metastatic pancreatic cancer |
| 1083 | Control | 94 | F | 1166 | 43.25 | I | Absent | Pneumonia, frailty of old age |
| 1092 | Control | 86 | F | 1288 | 36.25 | I | Moderate CAA | Mucinous adenocarcinoma of appendix |
| 32 | VaD | 84 | F | 1230 | 20 | Not specified | Not specified | Not specified^†^ |
| 92 | VaD | 72 | M | 1460 | 41 | Not specified | Not specified | Not specified^†^ |
| 131 | VaD | 86 | F | 1060 | 28 | Not specified | Not specified | Not specified^†^ |
| 170 | VaD | 90 | F | 1150 | 31 | Not specified | Not specified | Not specified^†^ |
| 232 | VaD | 89 | M | 1330 | 30 | Not specified | Not specified | Pulmonary embolism, ischaemic heart disease, old age |
| 347 | VaD | 76 | M | 1094 | 40 | Not specified | Not specified | Not specified^†^ |
| 787 | VaD | 78 | F | 1161 | 54 | 0 | Not specified | Vascular dementia |
| 931 | VaD | 76 | F | 1293 | 50 | II | Moderate CAA | Urosepsis |
| 1008 | VaD | 87 | M | 1160 | 34.25 | I | Not specified | Old age, vascular dementia, leg ulcer, type-2 diabetes |
| 1105 | VaD | 98 | F | 1169 | 22.75 | III | Severe CAA | Vascular dementia |
| For CAA pathology, ‘Not specified’ indicates that CAA diagnostic data were not included for that case in the brain bank database, whereas, ‘Absent’ indicates that CAA was measured but not identified in that sample. Abbreviations: CAA; Cerebral amyloid angiopathy; COPD: Chronic obstructive pulmonary disease; PMD: *Post-mortem* delay; UTI; Urinary tract infection; wt: Weight. ^†^Those patients in the VaD Class whose cause of death was not specified in the database had VaD as determined by post-mortem examination ut not specified at the time of writing as a cause of death in the SWDBB database. ^*^Cause of death as specified by the SWDBB database. | | | | | | | | |

| Supplementary Table 2. SWDBB multiregional individual patient characteristics (excluding the hippocampal cohort) | | | | | | | | |
| --- | --- | --- | --- | --- | --- | --- | --- | --- |
| Code | Class | Age at death | Sex | Brain wt (g) | PMD (h) | Braak stage | CAA | Cause of Death^*^ |
| 781 | Control | 87 | M | 1364 | 24 | II | Mild CAA | Acute renal failure, myeloma |
| 1092 | Control | 86 | F | 1288 | 36.25 | II | Moderate CAA | Mucinous adenocarcinoma of appendix |
| 786 | Control | 85 | M | 1337 | 30.5 | II | Not specified | Acute myocardial infarction due to ischaemic heart disease |
| 930 | Control | 94 | F | 1218 | 29.5 | II | Moderate CAA | Oesophageal carcinoma, ischaemic heart disease |
| 943 | Control | 70 | F | 1032 | 33.25 | II | Absent | Carcinomatosis, metastatic squamous cell carcinoma of larynx |
| 948 | Control | 82 | F | 1135 | 36 | II | Mild CAA | Carcinomatosis, ovarian/peritoneal carcinoma |
| 949 | Control | 69 | M | 1132 | 31.25 | II | Absent | Non-small cell lung cancer |
| 957 | Control | 86 | M | 1345 | 44.25 | II | Mild CAA | Infective exacerbation of COPD |
| 1067 | Control | 69 | F | 1121 | 38.25 | I | Absent | Metastatic pancreatic cancer |
| 1083 | Control | 94 | F | 1166 | 43.25 | I | Absent | Pneumonia, frailty of old age |
| 32 | VaD | 84 | F | 1230 | 20 | Not specified | Not specified | Not specified^†^ |
| 92 | VaD | 72 | M | 1460 | 20 | Not specified | Not specified | Not specified^†^ |
| 131 | VaD | 86 | F | 1060 | 28 | Not specified | Not specified | Not specified^†^ |
| 170 | VaD | 90 | F | 1150 | 31 | Not specified | Not specified | Not specified^†^ |
| 232 | VaD | 89 | M | 1330 | 30 | Not specified | Not specified | Pulmonary embolism, ischaemic heart disease, old age |
| 347 | VaD | 76 | M | 1094 | 40 | Not specified | Not specified | Not specified |
| 849 | VaD | 90 | M | 1262 | 45 | II | Mild CAA | Dementia^†^ |
| 931 | VaD | 76 | F | 1293 | 50 | II | Moderate CAA | Urosepsis |
| 787 | VaD | 78 | F | 1161 | 54 | 0 | Not specified | Vascular dementia |
| 1105 | VaD | 98 | F | 1169 | 22.75 | III | Severe CAA | Vascular dementia |
| For CAA pathology, ‘Not specified’ indicates that CAA diagnostic data were not included for that case in the brain bank database, whereas, ‘Absent’ indicates that CAA was measured but not identified in that sample. Abbreviations: CAA; Cerebral amyloid angiopathy; COPD: Chronic obstructive pulmonary disease; PMD: *Post-mortem* delay; UTI; Urinary tract infection; wt: Weight. ^†^Those patients in the VaD Class whose cause of death was not specified in the database had VaD as determined by post-mortem examination ut not specified at the time of writing as a cause of death in the SWDBB database. ^*^Cause of death as specified by the SWDBB database. | | | | | | | | |

| Supplementary Table 3. Group characteristics for the hippocampal cohort | | |
| --- | --- | --- |
| Variable | Control | VaD |
| Number | 10 | 10 |
| Age | 82 (9) | 84 (8) |
| Male sex, *n* (%) | 4 (40) | 4 (40) |
| *Post-mortem* delay (h) | 35.7 (6.6) | 35.1 (11.1) |
| Brain wt (g) | 1245 (135) | 1211 (120) |
| Wet-wt/dry-wt | 5.67 (0.65) | 5.94 (0.87) |
| Values are: age, *post-mortem* delay and brain wt, mean (SD); wet-wt/dry-wt ratio, mean  (SD) averaged across all samples. All differences were non-significant. | | |

| Supplementary Table 4. ICP-MS calibration standard solutions | | |
| --- | --- | --- |
| Internal standard solution | Solution | |
| 100 μg/L internal standard | 10 mL 2% nitric acid solution | 100 μL environmental calibration standard mixture |
| 50 μg/L internal standard | 15 mL 2% nitric acid solution | 75 μL environmental calibration standard mixture |
| 20 μg/L internal standard | 4 mL 2% nitric acid solution | 1 mL 100 μg/L solution |
| 10 μg/L internal standard | 9 mL 2% nitric acid solution | 1 mL 100 μg/L solution |
| 5 μg/L internal standard | 9 mL 2% nitric acid solution | 1 mL 50 μg/L solution |
| 2 μg/L internal standard | 4 mL 2% nitric acid solution | 1 mL 10 μg/L solution |
| 1 μg/L internal standard | 9 mL 2% nitric acid solution | 1 mL 10 μg/L solution |
| 0.5 μg/L internal standard | 9 mL 2% nitric acid solution | 1 mL 5 μg/L solution |
| Blank | 10 mL 2% nitric acid solution | - |

| Supplementary Table 5. Coefficient of variations for wet-weight ICP-MS analysis | | | | | | | | | | | | | | |
| --- | --- | --- | --- | --- | --- | --- | --- | --- | --- | --- | --- | --- | --- | --- |
| **Element** | **Cingulate gyrus** | | **Frontal Gyrus** | | **Thalamus** | | **Basal ganglia** | | **Middle temporal gyrus** | | **Occipital cortex** | | **Hippocampus** | |
|  | Control | VaD | Control | VaD | Control | VaD | Control | VaD | Control | VaD | Control | VaD | Control | VaD |
| Na | 11.49 | 11.03 | 12.63 | 10.98 | 13.67 | 27.57 | 18.29 | 18.87 | 12.75 | 10.34 | 25.42 | 18.24 | 11.49 | 14.47 |
| Mg | 13.47 | 7.251 | 7.751 | 6.983 | 18.23 | 14.31 | 6.133 | 3.655 | 10.46 | 12.16 | 15.32 | 12.25 | 7.518 | 10.46 |
| K | 7.715 | 13.80 | 10.33 | 8.254 | 24.01 | 20.28 | 10.29 | 8.717 | 11.00 | 13.12 | 18.22 | 16.44 | 12.24 | 15.98 |
| Ca | 36.94 | 34.82 | 102.5 | 12.70 | 24.14 | 58.36 | 18.23 | 44.21 | 203.8 | 139.4 | 20.12 | 44.03 | 28.01 | 179.8 |
| Mn | 14.45 | 8.253 | 14.19 | 10.89 | 18.93 | 23.40 | 21.87 | 39.29 | 11.96 | 12.93 | 43.04 | 14.88 | 17.38 | 66.54 |
| Fe | 20.03 | 18.48 | 11.80 | 121.7 | 31.83 | 21.00 | 44.11 | 64.34 | 23.77 | 17.51 | 69.62 | 49.56 | 21.48 | 29.16 |
| Cu | 21.30 | 26.38 | 20.67 | 26.61 | 30.05 | 37.30 | 22.68 | 19.32 | 24.95 | 27.00 | 30.68 | 18.45 | 23.11 | 19.37 |
| Zn | 19.13 | 11.81 | 11.43 | 7.489 | 11.92 | 15.69 | 16.18 | 17.94 | 15.07 | 8.039 | 16.98 | 8.772 | 12.23 | 14.59 |
| Se | 13.15 | 75.22 | 15.23 | 80.89 | 20.20 | 66.51 | 13.10 | 25.51 | 16.68 | 73.43 | 15.08 | 72.69 | 13.19 | 13.69 |

| Supplementary Table 6. Coefficient of variations for dry-weight ICP-MS analysis | | | | | | | | | | | | | | |
| --- | --- | --- | --- | --- | --- | --- | --- | --- | --- | --- | --- | --- | --- | --- |
| **Element** | **Cingulate gyrus** | | **Frontal Gyrus** | | **Thalamus** | | **Basal ganglia** | | **Middle temporal gyrus** | | **Occipital cortex** | | **Hippocampus** | |
|  | Control | VaD | Control | VaD | Control | VaD | Control | VaD | Control | VaD | Control | VaD | Control | VaD |
| Na | 22.78 | 20.01 | 22.24 | 22.70 | 34.31 | 48.50 | 38.28 | 29.91 | 24.59 | 37.18 | 29.18 | 38.75 | 19.97 | 26.13 |
| Mg | 12.76 | 10.69 | 5.357 | 7.482 | 10.06 | 17.21 | 17.36 | 16.40 | 17.87 | 35.03 | 7.765 | 8.255 | 7.035 | 7.739 |
| K | 21.17 | 23.39 | 9.931 | 10.48 | 10.12 | 12.07 | 20.26 | 16.22 | 20.27 | 43.83 | 14.22 | 14.61 | 12.92 | 14.45 |
| Ca | 30.48 | 37.57 | 74.44 | 95.64 | 197.5 | 36.31 | 206.8 | 109.1 | 35.49 | 84.74 | 27.47 | 40.58 | 26.87 | 29.35 |
| Mn | 21.71 | 14.35 | 84.84 | 16.85 | 13.00 | 23.32 | 36.42 | 108.1 | 20.07 | 45.04 | 22.21 | 17.98 | 17.02 | 29.59 |
| Fe | 14.60 | 17.48 | 16.25 | 105.7 | 15.23 | 33.87 | 42.77 | 57.08 | 19.90 | 29.37 | 9.245 | 52.55 | 21.47 | 19.07 |
| Cu | 29.86 | 32.92 | 19.00 | 26.93 | 28.76 | 28.62 | 28.22 | 25.02 | 33.29 | 22.87 | 28.10 | 25.73 | 19.29 | 18.89 |
| Zn | 24.97 | 25.91 | 11.36 | 14.71 | 16.36 | 32.95 | 26.20 | 30.60 | 20.50 | 34.10 | 16.23 | 19.95 | 16.98 | 17.70 |
| Se | 18.93 | 51.29 | 8.939 | 71.22 | 17.09 | 41.59 | 21.69 | 28.84 | 18.97 | 135.3 | 16.10 | 42.35 | 9.962 | 11.97 |

| Supplementary Table 7. Mean coefficient of variations for multiregional wet- and dry-weight ICP-MS analysis | | |
| --- | --- | --- |
| Element | Wet-weight CV | Dry-weight CV |
| Na | **15.52** | 29.61 |
| Mg | **10.43** | 12.93 |
| K | **13.60** | 17.42 |
| Ca | **67.65** | 73.74 |
| Mn | **22.71** | 33.61 |
| Fe | 38.89 | **32.47** |
| Cu | **24.85** | 26.25 |
| Zn | **13.38** | 22.04 |
| Se | 36.76 | **35.30** |
| Values in bold represent the lowest mean CV for that element across all regions analysed. | | |

| Supplementary Table 8. Dry-weight metal concentrations in the middle temporal gyrus of VaD and control brains | | | | | |
| --- | --- | --- | --- | --- | --- |
| Element | Units | Reference isotope | Control | VaD | *p*-value |
| Na | mmol/kg | ^23^Na | 478 (409-542) | 523 (485-735) | 0.2176 |
| Mg | mmol/kg | ^23^Mg | 28 (26-31) | 29 (27-31) | 0.5288 |
| K | mmol/kg | ^39^K | 394 (344-466) | 395 (335-425) | 0.7959 |
| Ca | mmol/kg | ^44^Ca | 10.71 (6.83-12.28) | 16.00 (8.50-24.74) | 0.1051 |
| Mn | µmol/kg | ^55^Mn | 19 (17-24) | 20 (19-22) | 0.4359 |
| Fe | mmol/kg | ^56^Fe | 5.00 (4.25-5.39) | 5.28 (4.75-5.72) | 0.3527 |
| Cu | µmol/kg | ^63^Cu | 294 (249-383) | 363 (324-474) | 0.0892 |
| Zn | µmol/kg | ^66^Zn | 1227 (1040-1350) | 1225 (1166-1322) | 0.6842 |
| Se | µmol/kg | ^78^Se | 10.61 (9.32-11.28) | 11.45 (10.83-12.38) | 0.0892 |
| Data are medians (interquartile range); *p*-values for significance of between-group differences were calculated by Mann-Whitney U test based on dry-weight measurements from control (n = 10) and VaD (n = 10) brains. | | | | | |

| Supplementary Table 9. Dry-weight metal concentrations in the thalamus of VaD and control brains | | | | | |
| --- | --- | --- | --- | --- | --- |
| Element | Units | Reference isotope | Control | VaD | *p*-value |
| Na | mmol/kg | ^23^Na | 384 (132) | 405 (196) | 0.7798 |
| Mg | mmol/kg | ^23^Mg | 25 (3) | 24 (4) | 0.3313 |
| K | mmol/kg | ^39^K | 315 (32) | 279 (34) | **0.0237** |
| Ca | mmol/kg | ^44^Ca | 23.39 (46.19) | 7.88 (2.86) | 0.3165 |
| Mn | µmol/kg | ^55^Mn | 30 (4) | 28 (7) | 0.6314 |
| Fe | mmol/kg | ^56^Fe | 3.88 (0.59) | 3.93 (1.33) | 0.9105 |
| Cu | µmol/kg | ^63^Cu | 223 (64) | 265 (76) | 0.1964 |
| Zn | µmol/kg | ^66^Zn | 1000 (164) | 927 (305) | 0.5179 |
| Se | µmol/kg | ^78^Se | 10.12 (1.73) | 11.51 (4.79) | 0.4077 |
| Data are means (SD); *p*-values for significance of between-group differences were calculated by Welch’s t-test based on dry-weight measurements from control (n = 10) and VaD (n = 10) brains. Significant values (*p* < 0.05) are shown in bold. | | | | | |

| Supplementary Table 10. Dry-weight metal concentrations in the basal ganglia of VaD and control brains | | | | | |
| --- | --- | --- | --- | --- | --- |
| Element | Units | Reference isotope | Control | VaD | *p*-value |
| Na | mmol/kg | ^23^Na | 254 (97) | 357 (107) | **0.0375** |
| Mg | mmol/kg | ^23^Mg | 25 (4) | 26 (4) | 0.7399 |
| K | mmol/kg | ^39^K | 379 (77) | 392 (64) | 0.6944 |
| Ca | mmol/kg | ^44^Ca | 6.69 (2.03) | 8.79 (3.43) | 0.1374 |
| Mn | µmol/kg | ^55^Mn | 46 (17) | 68 (74) | 0.3641 |
| Fe | mmol/kg | ^56^Fe | 16.85 (7.21) | 16.66 (9.51) | 0.9606 |
| Cu | µmol/kg | ^63^Cu | 374 (106) | 490 (123) | **0.0370** |
| Zn | µmol/kg | ^66^Zn | 1073 (281) | 1204 (368) | 0.3846 |
| Se | µmol/kg | ^78^Se | 11.86 (2.57) | 13.25 (3.82) | 0.3534 |
| Data are means (SD); *p*-values for significance of between-group differences were calculated by Welch’s t-test test based on dry-weight measurements from control (n = 10) and VaD (n = 10) brains. Significant values (*p* < 0.05) are shown in bold. | | | | | |

| Supplementary Table 11. Dry-weight metal concentrations in the occipital cortex of VaD and control brains | | | | | |
| --- | --- | --- | --- | --- | --- |
| Element | Units | Reference isotope | Control | VaD | *p*-value |
| Na | mmol/kg | ^23^Na | 381 (111) | 441 (171) | 0.3690 |
| Mg | mmol/kg | ^23^Mg | 24 (2) | 23 (2) | 0.2721 |
| K | mmol/kg | ^39^K | 356 (51) | 323 (47) | 0.1469 |
| Ca | mmol/kg | ^44^Ca | 8.69 (2.39) | 8.89 (3.61) | 0.8887 |
| Mn | µmol/kg | ^55^Mn | 20.01 (4.45) | 19.78 (3.56) | 0.9014 |
| Fe | mmol/kg | ^56^Fe | 5.40 (0.50) | 6.28 (3.30) | 0.4290 |
| Cu | µmol/kg | ^63^Cu | 319 (90) | 353 (91) | 0.4044 |
| Zn | µmol/kg | ^66^Zn | 1040 (169) | 1047 (209) | 0.9362 |
| Se | µmol/kg | ^78^Se | 11.77 (1.90) | 14.18 (6.01) | 0.2520 |
| Data are means (SD); *p*-values for significance of between-group differences were calculated by Welch’s t-test based on dry-weight measurements from control (n = 10) and VaD (n = 10) brains. | | | | | |

| Supplementary Table 12. Dry-weight metal concentrations in the frontal gyrus of VaD and control brains | | | | | |
| --- | --- | --- | --- | --- | --- |
| Element | Units | Reference isotope | Control | VaD | *p*-value |
| Na | mmol/kg | ^23^Na | 462 (103) | 645 (146) | **0.0052** |
| Mg | mmol/kg | ^23^Mg | 26 (1) | 28 (2) | 0.0680 |
| K | mmol/kg | ^39^K | 333 (33) | 358 (38) | 0.1348 |
| Ca | mmol/kg | ^44^Ca | 12.25 (9.12) | 12.99 (12.42) | 0.8815 |
| Mn | µmol/kg | ^55^Mn | 27 (27) | 21 (4) | 0.4484 |
| Fe | mmol/kg | ^56^Fe | 5.21 (0.85) | 7.55 (7.98) | 0.3790 |
| Cu | µmol/kg | ^63^Cu | 307 (58) | 399 (107) | **0.0317** |
| Zn | µmol/kg | ^66^Zn | 1085 (123) | 1150 (169) | 0.3400 |
| Se | µmol/kg | ^78^Se | 11.14 (1.00) | 16.05 (11.43) | 0.2086 |
| Data are means (SD); *p*-values for significance of between-group differences were calculated by Welch’s t-test based on dry-weight measurements from control (n = 10) and VaD (n = 10) brains. Significant values (*p* < 0.05) are shown in bold. | | | | | |

| Supplementary Table 13. Dry-weight metal concentrations in the cingulate gyrus of VaD and control brains | | | | | |
| --- | --- | --- | --- | --- | --- |
| Element | Units | Reference isotope | Control | VaD | *p*-value |
| Na | mmol/kg | ^23^Na | 398 (91) | 453 (91) | 0.1871 |
| Mg | mmol/kg | ^23^Mg | 24 (3) | 24 (3) | 0.6851 |
| K | mmol/kg | ^39^K | 370 (78) | 309 (72) | 0.0846 |
| Ca | mmol/kg | ^44^Ca | 10.08 (3.07) | 8.72 (3.27) | 0.3486 |
| Mn | µmol/kg | ^55^Mn | 21 (5) | 21 (3) | 0.9089 |
| Fe | mmol/kg | ^56^Fe | 3.61 (0.53) | 3.64 (0.64) | 0.8980 |
| Cu | µmol/kg | ^63^Cu | 269 (80) | 307 (101) | 0.3724 |
| Zn | µmol/kg | ^66^Zn | 1119 (280) | 1042 (270) | 0.5374 |
| Se | µmol/kg | ^78^Se | 10.35 (1.96) | 12.78 (6.56) | 0.2849 |
| Data are means (SD); *p*-values for significance of between-group differences were calculated by Welch’s t-test based on dry-weight measurements from control (n = 10) and VaD (n = 10) brains. | | | | | |

| Supplementary Table 14. Dry-weight metal concentrations in the hippocampus of VaD and control brains | | | | | |
| --- | --- | --- | --- | --- | --- |
| Element | Units | Reference isotope | Control | VaD | *p*-value |
| Na | mmol/kg | ^23^Na | 471 (94) | 609 (159) | **0.0326** |
| Mg | mmol/kg | ^23^Mg | 28 (2) | 29 (2) | 0.2673 |
| K | mmol/kg | ^39^K | 321 (42) | 304 (44) | 0.3964 |
| Ca^†^ | mmol/kg | ^44^Ca | 11.26 (3.03) | 12.36 (3.63) | 0.4858 |
| Mn | µmol/kg | ^55^Mn | 28 (28) | 29 (29) | 0.5408 |
| Fe | mmol/kg | ^56^Fe | 4.10 (0.88) | 4.25 (0.81) | 0.6834 |
| Cu | µmol/kg | ^63^Cu | 279 (54) | 334 (63) | 0.0518 |
| Zn | µmol/kg | ^66^Zn | 1278 (217) | 1514 (268) | **0.0447** |
| Se | µmol/kg | ^78^Se | 9.81 (0.98) | 12.27 (6.68) | 0.2769 |
| Data are means (SD); *p*-values for significance of between-group differences were calculated by Welch’s t-test based on dry-weight measurements from control (n = 10) and VaD (n = 10) brains. Significant values (*p* < 0.05) are shown in bold. ^†^One sample in the Ca analysis was identified as an outlier and removed from analysis. | | | | | |

| Supplementary Table 15. Global brain post-hoc power analysis | | |
| --- | --- | --- |
| Element | Wet-weight tissue | Dry-weight tissue |
| Na | **0.999** | **0.961** |
| Mg | 0.101 | 0.146 |
| K | 0.776 | 0.112 |
| Ca | 0.172 | 0.051 |
| Mn | 0.081 | 0.114 |
| Fe | 0.249 | 0.091 |
| Cu | 0.448 | **0.959** |
| Zn | 0.050 | 0.273 |
| Se | 0.779 | 0.684 |
| Values highlighted in bold satisfy the statistical power of > 80%. Post-hoc power analyses were generated using an α error probability of 0.05. Values were determined using G*Power (v. 3.1.9.4). | | |

| Supplementary Table 16. Multiregional *post-hoc* power estimates | | | | | | | | | | |
| --- | --- | --- | --- | --- | --- | --- | --- | --- | --- | --- |
| Brain region | Tissue condition | Na | Mg | K | Ca | Mn | Fe | Cu | Zn | Se |
| Cingulate gyrus | Dry-wt | 0.255 | 0.067 | 0.408 | 0.149 | 0.051 | 0.052 | 0.140 | 0.466 | 0.188 |
|  | Wet-wt | **0.989** | 0.096 | 0.598 | 0.052 | 0.073 | 0.102 | 0.417 | 0.050 | 0.219 |
| Frontal gyrus | Dry-wt | **0.862** | 0.224 | 0.318 | 0.052 | 0.116 | 0.141 | 0.618 | 0.157 | 0.187 |
|  | Wet-wt | **0.961** | 0.067 | 0.134 | 0.236 | 0.050 | 0.145 | 0.469 | 0.062 | 0.187 |
| Thalamus | Dry-wt | 0.058 | 0.159 | 0.646 | 0.171 | 0.087 | 0.050 | 0.246 | 0.096 | 0.128 |
|  | Wet-wt | 0.354 | 0.297 | 0.073 | 0.078 | 0.055 | 0.200 | 0.241 | 0.297 | 0.297 |
| Basal ganglia | Dry-wt | 0.566 | 0.062 | 0.067 | 0.352 | 0.147 | 0.050 | 0.570 | 0.135 | 0.181 |
|  | Wet-wt | 0.745 | 0.228 | 0.236 | **0.9998** | 0.060 | 0.192 | 0.428 | 0.060 | 0.181 |
| Middle temporal gyrus | Dry-wt | 0.426 | 0.212 | 0.288 | 0.071 | 0.188 | 0.227 | 0.361 | 0.186 | 0.186 |
|  | Wet-wt | 0.619 | 0.085 | 0.288 | 0.071 | 0.188 | 0.227 | 0.361 | 0.186 | 0.186 |
| Occipital cortex | Dry-wt | 0.142 | 0.190 | 0.299 | 0.052 | 0.052 | 0.123 | 0.128 | 0.051 | 0.208 |
|  | Wet-wt | **0.882** | 0.132 | 0.328 | 0.533 | **0.876** | 0.675 | 0.185 | 0.211 | 0.119 |
| Hippocampus | Dry-wt | 0.608 | 0.194 | 0.13 | 0.107 | 0.091 | 0.068 | 0.506 | 0.535 | 0.113 |
|  | Wet-wt | **0.922** | 0.080 | 0.776 | 0.178 | 0.100 | 0.170 | 0.061 | 0.052 | 0.170 |
| Values highlighted in bold satisfy the statistical power of > 80%. Post-hoc power analyses were generated using an α error probability of 0.05. Values were determined using G*Power (v. 3.1.9.4). | | | | | | | | | | |

| Supplementary Table 17. Multiregional sample size estimates | | | | | | | | | | |
| --- | --- | --- | --- | --- | --- | --- | --- | --- | --- | --- |
| Brain region | Tissue condition | Na | Mg | K | Ca | Mn | Fe | Cu | Zn | Se |
| Cingulate gyrus | Dry-wt | 86 | 936 | 50 | 174 | 12492 | 9282 | 190 | 42 | 126 |
|  | Wet-wt | **12** | 366 | 32 | 6968 | 700 | 322 | 48 | 332880 | 104 |
| Frontal gyrus | Dry-wt | **18** | 102 | 66 | 6810 | 254 | 186 | 30 | 160 | 88 |
|  | Wet-wt | **14** | 956 | 204 | 94 | 1426264 | 180 | 42 | 1398 | 126 |
| Thalamus | Dry-wt | 1936 | 158 | 28 | 142 | 444 | 116272 | 90 | 360 | 216 |
|  | Wet-wt | 60 | 1778 | 720 | 25820 | 3358 | 3912 | 92 | 1778 | 152 |
| Basal ganglia | Dry-wt | 34 | 1778 | 720 | 25820 | 3358 | 3912 | 92 | 1778 | 152 |
|  | Wet-wt | 24 | 98 | 94 | **8** | 1634 | 122 | 48 | 1646 | 132 |
| Middle temporal gyrus | Dry-wt | 48 | 108 | 74 | 784 | 126 | 100 | 58 | 128 | 128 |
|  | Wet-wt | 30 | 474 | 100 | 432 | 100 | 92 | 66 | 21238 | 36 |
| Occipital cortex | Dry-wt | 186 | 124 | 72 | 7724 | 9622 | 232 | 218 | 23088 | 110 |
|  | Wet-wt | **18** | 208 | 64 | 36 | **18** | 28 | 128 | 108 | 244 |
| Hippocampus | Dry-wt | 32 | 122 | 210 | 292 | 402 | 914 | 40 | 36 | 268 |
|  | Wet-wt | **16** | 546 | 22 | 136 | 332 | 144 | 1472 | 7608 | 144 |
| Values in bold represent samples sizes that are below n = 20. Required samples sizes were generated using a statistical power of 80% and an α error probability of 0.05. Values were determined using G*Power (v. 3.1.9.4). | | | | | | | | | | |

| Supplementary Table 18. Global brain Na/K ratio comparisons between VaD and control brain tissue | | | | |
| --- | --- | --- | --- | --- |
| Class | Na (mmol/kg) | K (mmol/kg) | Na/K ratio | *p*-value |
| **Wet-weight tissue** | | | | |
| Control | 73 (61-83) | 64 (57-76) | 1.16 | <0.0001 |
| VaD | 91 (80-98) | 59 (53-70) | 1.58 |  |
| **Dry-weight tissue** | | | | |
| Control | 410 (314-507) | 340 (307-399) | 1.15 | 0.0001 |
| VaD | 494 (384-596) | 321 (289-287) | 1.57 |  |
| Data are medians (interquartile range); *p*-values for significance of between-group global brain Na/K ratio differences were calculated by Mann-Whitney U based on measurements from control (n = 70) and VaD (n = 70) brains. | | | | |

| Supplementary Table 19. Wet-weight Na/K ratio comparisons between VaD and control brain tissue | | | | |
| --- | --- | --- | --- | --- |
| Class | Na (mmol/kg) | K (mmol/kg) | Na/K ratio | *p*-value |
| **Thalamus** | | | | |
| Control | 72 (10) | 65 (16) | 1.20 | 0.4813 |
| VaD | 85 (23) | 62 (13) | 1.48 |  |
| **Basal ganglia** | | | | |
| Control | 55 (10) | 80 (8) | 0.72 | **0.0039** |
| VaD | 70 (13) | 75 (7) | 0.94 |  |
| **Cingulate gyrus** | | | | |
| Control | 70 (8) | 62 (5) | 1.14 | **0.0003** |
| VaD | 88 (10) | 56 (8) | 1.62 |  |
| **Frontal gyrus** | | | | |
| Control | 82 (10) | 58 (6) | 1.43 | **0.0089** |
| VaD | 101 (11) | 56 (5) | 1.81 |  |
| **Middle temporal gyrus** | | | | |
| Control | 83 (11) | 67 (7) | 1.26 | 0.0657 |
| VaD | 93 (10) | 62 (8) | 1.53 |  |
| **Occipital cortex** | | | | |
| Control | 64 (16) | 74 (13) | 0.94 | 0.0524 |
| VaD | 88 (16) | 65 (11) | 1.44 |  |
| **Hippocampus** | | | | |
| Control | 78 (10) | 56 (7) | 1.43 | **0.0039** |
| VaD | 97 (14) | 47 (7) | 2.15 |  |
| Data are means (SD); *p*-values for significance of between-group Na/K ratio differences were calculated by Mann-Whitney U based on measurements from control (n = 10) and VaD (n = 10) brains. Significant values (*p* < 0.05) are shown in bold. | | | | |

| Supplementary Table 20. Dry-weight Na/K ratio comparisons between VaD and control brain tissue | | | | |
| --- | --- | --- | --- | --- |
| Class | Na (mmol/kg) | K (mmol/kg) | Na/K ratio | *p*-value |
| **Thalamus** | | | | |
| Control | 384 (132) | 315 (32) | 1.22 | 0.3957 |
| VaD | 405 (196) | 279 (34) | 1.44 |  |
| **Basal ganglia** | | | | |
| Control | 254 (97) | 379 (77) | 0.66 | **0.0106** |
| VaD | 357 (107) | 392 (64) | 0.91 |  |
| **Cingulate gyrus** | | | | |
| Control | 398 (91) | 370 (78) | 1.09 | **0.0012** |
| VaD | 453 (91) | 309 (72) | 1.50 |  |
| **Frontal gyrus** | | | | |
| Control | 462 (103) | 333 (33) | 1.40 | **0.0157** |
| VaD | 645 (146) | 358 (38) | 1.79 |  |
| **Middle temporal gyrus** | | | | |
| Control | 462 (114) | 394 (80) | 1.18 | 0.0613 |
| VaD | 612 (223) | 434 (190) | 1.46 |  |
| **Occipital cortex** | | | | |
| Control | 381 (111) | 356 (51) | 1.07 | 0.1780 |
| VaD | 441 (171) | 323 (47) | 1.40 |  |
| **Hippocampus** | | | | |
| Control | 471 (94) | 321 (42) | 1.49 | **0.0314** |
| VaD | 609 (159) | 304 (44) | 2.04 |  |
| Data are means (SD); *p*-values for significance of between-group Na/K ratio differences were calculated by Welch’s t-tests based on measurements from control (n = 10) and VaD (n = 10) brains. Significant values (*p* < 0.05) are shown in bold. | | | | |

| Supplementary Table 21. Dry-weight Na/K ratio comparisons between AD and control brain tissue | | | | |
| --- | --- | --- | --- | --- |
| Class | Na (mmol/kg) | K (mmol/kg) | Na/K ratio | *p*-value |
| **Cingulate gyrus (Control [n = 8]; AD [n = 9])** | | | | |
| Control | 523 (151) | 550 (89) | 0.96 | 0.6408 |
| AD | 460 (185) | 449 (55) | 1.03 |  |
| **Middle temporal gyrus (Control [n = 9]; AD [n = 9])** | | | | |
| Control | 319 (86) | 413 (64) | 0.76 | **0.0011** |
| AD | 566 (148) | 373 (53) | 1.54 |  |
| **Hippocampus (Control [n = 9]; AD [n = 8])** | | | | |
| Control | 389 (82) | 323 (27) | 1.20 | **0.0006** |
| AD | 595 (91) | 321 (76) | 1.91 |  |
| Data are means (SD) taken from our previous investigation of AD (Xu et al., 2017); *p*-values for significance of between-group Na/K ratio differences were calculated by Welch’s t-tests based on measurements from control and AD brains. Significant values (*p* < 0.05) are shown in bold. | | | | |

**A**

**B**

**Supplementary Figure 1. Grand-mean Cu analysis from wet- and dry-weight post-mortem brain tissue.** Data are means (±95% CI) from both (A) dry- and (B) wet-weight Cu analyses. n = 140 (Controls = 70; VaD = 70) for each analysis.

Supplementary Figure 2. Simple linear regression plots between dry- and wet-weight sodium and copper levels in VaD brain tissue. Data represents simple linear regression analysis between grand-mean Na (n = 70) and K (n = 70) values from both (A) dry- and (B) wet-weight metal analysis. A single outlier was removed from the wet-weight dataset.

**A**

**B**


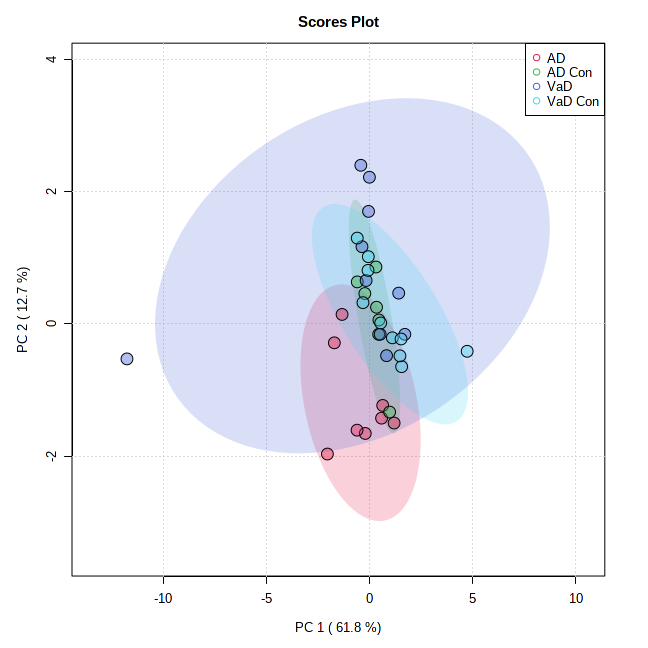


MTG


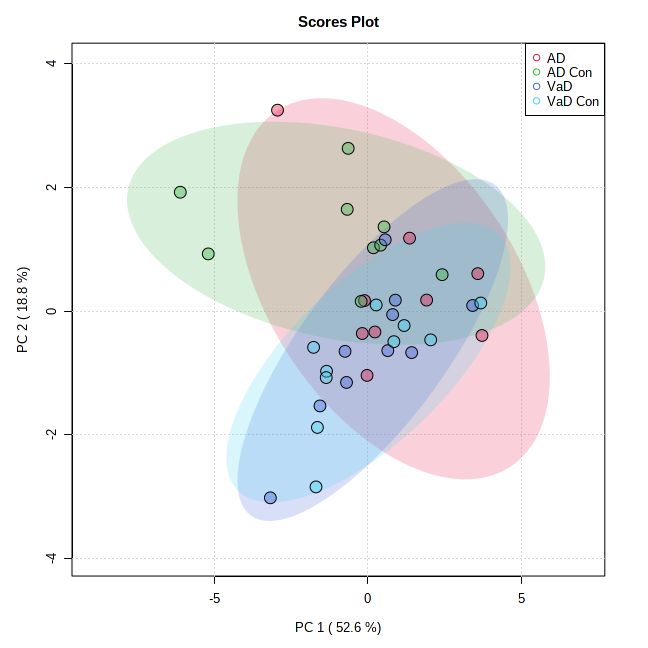


CG


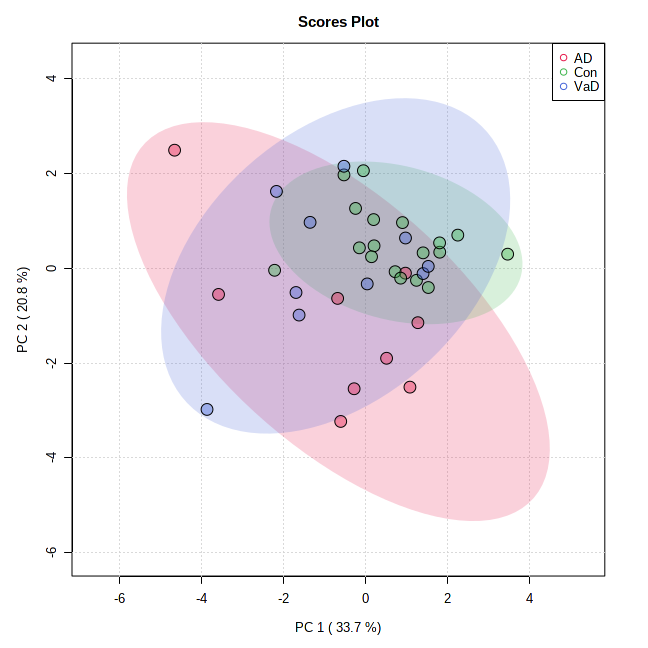


HP

**Supplementary Figure 3. Two dimensional PCA plots for human dry-weight post-mortem tissue from AD and VaD.** Data represents PCA plots using ICP-MS-metallomic data from VaD con (n = 10; light blue), AD con (n = 8; green), VaD (n = 10; dark blue), AD (n = 9; red) dry-weight post-mortem brain tissue from the CG and MTG. For the HP, data represents a PCA plot using ICP-MS-metallomic data from Con (n = 18; green), VaD (n = 10; blue), AD (n = 9; red) dry-weight post-mortem tissue. The coloured ellipses around each cohort signify 95% confidence regions. No visible separation was apparent between all cohorts. Abbreviations: AD: Alzheimer’s disease; Con: Control; ICP-MS: Inductively coupled plasma-mass spectrometry; VaD: Vascular dementia.


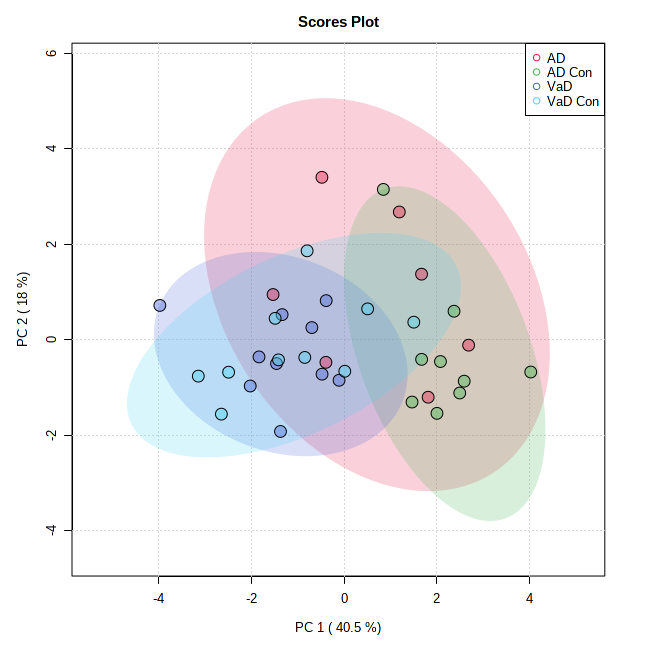

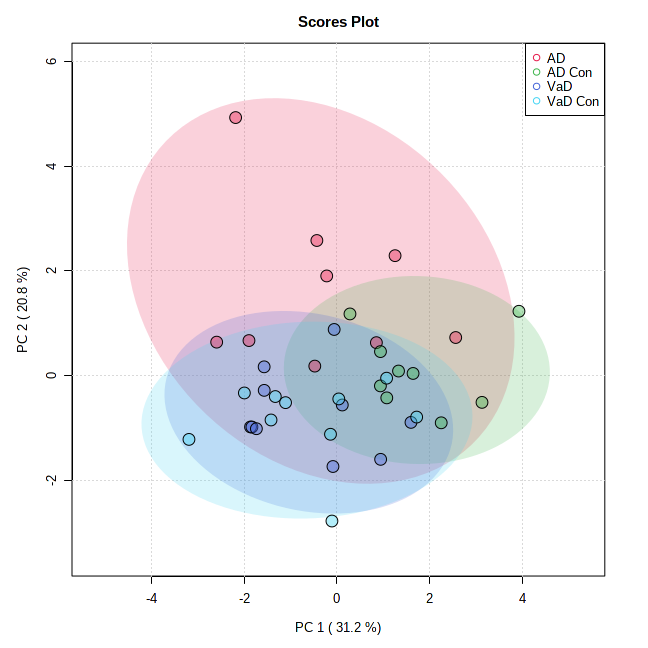

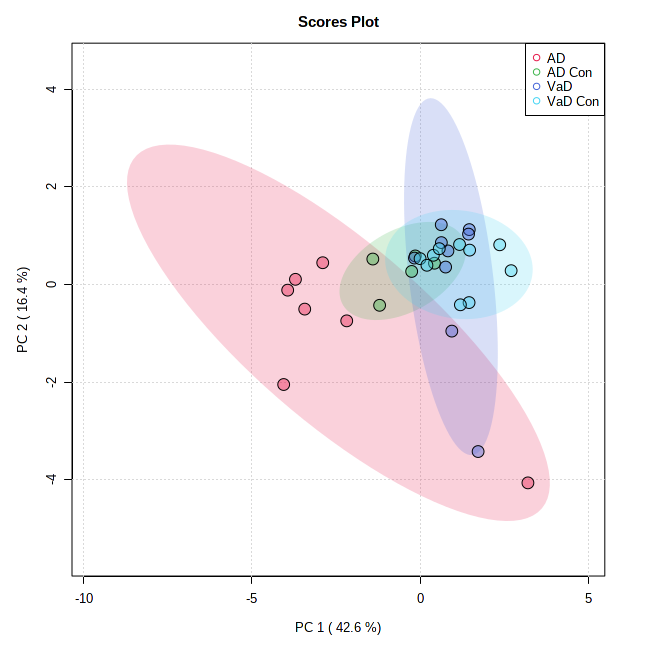


MTG

HP

CG

**Supplementary Figure 4. Two dimensional PCA plots for human wet-weight post-mortem tissue from AD and VaD.** Data represents PCA plots using ICP-MS-metallomic data from VaD con (n = 10; light blue), AD con (n = 5; green), VaD (n = 10; dark blue), AD (n = 7; red), dry-weight post-mortem brain tissue from the CG, MTG, and HP. The coloured ellipses around each cohort signify 95% confidence regions. No visible separation was apparent between all cohorts. Abbreviations: AD: Alzheimer’s disease; Con: Control; ICP-MS: Inductively coupled plasma-mass spectrometry; VaD: Vascular dementia.

**A**

**B**

**Supplementary Figure 5. Individual multiregional Na/K ratios for wet- and dry-weight post-mortem brain tissue.** Data are Na/K ratios (±95% CI) from (A) wet- and (B) dry-weight multiregional analyses (Control = 10; VaD = 10).
